# A Genomic Quantitative Study on the Contribution of the Ancestral-State Bases Relative to Derived Bases in the Divergence and Local Adaptation of *Populus davidiana*

**DOI:** 10.3390/genes14040821

**Published:** 2023-03-29

**Authors:** Dandan Zhao, Jianguo Zhang, Nan Hui, Li Wang, Yang Tian, Wanning Ni, Jinhua Long, Li Jiang, Yi Li, Songfeng Diao, Jinhua Li, Luke R. Tembrock, Zhiqiang Wu, Zhaoshan Wang

**Affiliations:** 1 State Key Laboratory of Tree Genetics and Breeding, Key Laboratory of Silviculture of the State Forestry Administration, Research Institute of Forestry, Chinese Academy of Forestry, Beijing 100091, China; 2 Collaborative Innovation Center of Sustainable, Forestry in Southern China, Nanjing Forestry University, Nanjing 210037, China; 3 Department of Agricultural Biology, Colorado State University, Fort Collins, CO 80523, USA; 4 Shenzhen Branch, Guangdong Laboratory for Lingnan Modern Agriculture, Genome Analysis Laboratory of the Ministry of Agriculture, Agricultural Genomics Institute at Shenzhen, Chinese Academy of Agricultural Sciences, Shenzhen 518120, China

**Keywords:** whole-genome re-sequencing, ancestral-state base (ASB), derived base (DB), linked selection, demographic histories

## Abstract

Identifying alleles associated with adaptation to new environments will advance our understanding of evolution from the molecular level. Previous studies have found that the *Populus davidiana* southwest population in East Asia has differentiated from other populations in the range. We aimed to evaluate the contributions of the ancestral-state bases (ASBs) relative to derived bases (DBs) in the local adaptation of *P. davidiana* in the Yunnan–Guizhou Plateau from a quantitative perspective using whole-genome re-sequencing data from 90 *P. davidiana* samples from three regions across the species range. Our results showed that the uplift of the Qinghai–Tibet Plateau during the Neogene and associated climate fluctuations during the Middle Pleistocene were likely an important factor in the early divergence of *P. davidiana*. Highly differentiated genomic regions between populations were inferred to have undergone strong linked natural selection, and ASBs are the chief means by which populations of *P. davidiana* adapt to novel environmental conditions; however, when adapting to regions with high environmental differences relative to the ancestral range, the proportion of DBs was significantly higher than that of background regions, as ASBs are insufficient to cope with these environments. Finally, a number of genes were identified in the outlier region.

## 1. Introduction

Elucidating the molecular mechanisms involved in adaptive evolution is a core scientific question in evolutionary biology [1,2,3]. In the process of lineage divergence, changes in environmental conditions can lead over time to detectable differences between populations [4]. Divergence will be initiated under a scenario with any of the following driving forces. For instance, genetic drift is extremely strong in the absence of gene flow in small populations experiencing a bottleneck and thus can invariably bring about differentiation in the absence of selection [5]. Demographic processes including population bottlenecks or expansions can accelerate or decrease the rate of differentiation of the genome through changes in the effective population sizes (*N*_e_) of nascent daughter species [6]. These two factors affect the whole genome [7,8], whereas natural selection only affects the genomic regions harboring the loci associated with selected traits [8]. Suppression of recombination can increase genetic differentiation not only by limiting gene flow among species but also by reducing diversity through linked selection [9]. In addition, differentiation will also occur in the presence of mutations [9,10,11,12]. Accordingly, disentangling different driving factors of genetic divergence in populations is beneficial to understand the adaptive evolution of the species.

As some evidence suggests, many species are widely distributed because they are able to evolve subpopulations adapted to local environment conditions; the evolution and consequent expansion of ecological amplitude cannot occur without the appropriate genetic variation [13,14]. There are two frequently studied and interrelated sources of genetic variation by which populations can adapt at the molecular level to changing environmental conditions. The first involves selection on ancestral-state bases (ASBs) already present in a population, resulting in changes in the frequency of the selected locus; the second involves the emergence of a novel or improved function through mutation (derived bases, DBs), and thereafter selection acts based on increases or decreases in fitness [15,16,17]. 

An ASB indicates the base state being the same as the ancestral population at the same site, where they have previously persisted while being neutral or slightly deleterious but shift to being beneficial as they experience novel environmental parameters such as those experienced during range expansion [18,19]. A number of case studies of ecologically relevant genes have pointed out that adaptation often occurs through variation in ASBs [20,21,22,23,24,25]. First, the frequency of potentially advantageous alleles that arises from ancestor is generally higher than that of DBs, resulting in increased rates of adaptation among populations [26]. Second, alleles from ancestors have in many cases been subjected to a “selective filter”, increasing the probability that large effect alleles will be advantageous [15]. Introgressive hybridization is another means by which ASBs can be increased in short periods of time and such patterns have been frequently documented among *Populus* species [27,28,29]. Conversely, introgression can in some instances result in maladapted offspring through outbreeding depression [30].

Despite the importance of ASBs in rapidly adapting to novel environments and stressors, DBs (with a base state of the same site different from the ancestor) are essential in long-term macroevolution when an ASB is insufficient for coping with extreme or novel environments [15,31,32]. As the source of genetic variation and the raw material for selection [33], neutral mutations in the population are unaffected by natural selection and are randomly preserved, spread, or disappear depending on genetic drift; the fate of most DBs in functional gene regions is to be removed via selection, as the effects are generally deleterious—although the efficiency of selection on these mutations is dependent on factors such as the recombination rate, mutation rate, epistasis, and demography [34,35]. Thus, the probability that a beneficial allele obtained from a single DB becomes fixed in a population is low compared to that for an ASB, and it is greatly dependent on the strength of selection and size of the population [15]. It is evident that adaptation from DBs is likely to require many generations to positively influence the population or species in which they evolve. Given the characteristic differences between ASBs and DBs, we hypothesized that ASBs play a major role in the adaptation to new habitats overall, but when adapting to regions with high environmental differences relative to the ancestral range, the proportion of DBs will be significantly higher, as ASBs will usually be insufficient to cope with extreme environmental pressures.

The genus *Populus* L. (Salicaceae) is a model species for genetics and tree breeding. The species *P. davidiana* is a common tree found throughout mountain forests of China, eastern Russia, Korea, and Mongolia. Based on phylogeographic studies, genetic diversity in *P. davidiana* is arrayed in a north to south pattern [36], suggesting latitudinal migration associated with postglacial colonization as has been described for other *Populus* species [37,38,39]. Some previous studies concluded that this *P. davidiana* differentiation was caused by different environments [36,40]. The advent of re-sequencing technologies allows us to perform a whole-genome scan to characterize the mutations according to single nucleotide polymorphism (SNP) loci and to explore the causes of genome differentiation and the role of genetic variation from different sources on species adaptation. 

Here, based on whole-genome re-sequencing data, we reconstructed demographic histories, simulated different divergence scenarios among three lineages of *P. davidiana*, and quantitatively calculated the proportion of the allele fixation from ASBs and DBs. Specifically, we sought to (i) determine the differentiation degree and demographic histories of *P. davidiana* across the species range; (ii) estimate the respective contributions of ASBs and DBs during adaptive evolution, and test the hypothesis that the highly differentiated regions in the genome typically contain a higher proportion of DBs to adapt to extreme environments which ASBs are insufficient to cope with; and (iii) identify a number of genes that may be selected under the high-altitude environment in the Yunnan–Guizhou Plateau. We expect ASBs to play a major role in adapting to new environments, but we predict that the contribution of DBs will also significantly increase in extreme environments.

## 2. Materials and Methods

### 2.1. Population Sampling, Sequencing, Quality Control, and Read Mapping

**Figure 1:**
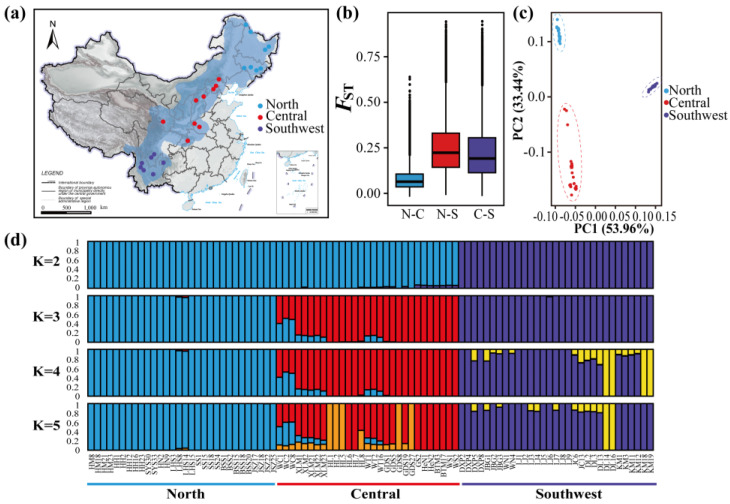
Sample collection, relative divergence measures (*F*_ST_), and population structure analysis of 90 *P. davidiana*. (**a**) Blue shadow represents the range extent of *P. davidiana*, 30 northern individuals (blue) were collected in Heilongjiang and Jilin of China, 29 central individuals (red) were collected in Hebei, Beijing, Shanxi, Gansu, Henan, and Chongqing, and 31 southwestern individuals (purple) were collected in Sichuan, Guizhou, and Yunnan (Yunnan–Guizhou Plateau). (**b**) Comparison of relative divergence measures (*F*_ST_) between north and central populations (N−C), between north and southwest populations (N−S), and between central and southwest populations (C−S). (**c**) Results from a PCA on the genetic covariance matrix for all individuals of northern individuals (blue circles) of *P. davidiana*, central individuals (red circles), and southwestern individuals (purple circles). (**d**) Population genetic structure in the samples based on an analysis using NGSadmix in ANGSD based on genotype likelihoods.

Leaf material (healthy, complete, clean, no pests and diseases) from a total of 90 individuals (diameter at breast height above 5 cm in each individual) of *P. davidiana* were collected across the geographical range in East Asia, of which 30 individuals were from northern East Asia, 29 in central East Asia, and 31 from southwest East Asia (Yunnan–Guizhou plateau) (Figure 1a; Table S1). Most of the sampling sites are located in mountainous areas, and the soil is slightly acidic to neutral. When samples were collected from the same area, a distance of at least 200 m between collections was used to avoid sampling clones. Samples were dried in silica gel to prevent DNA degradation. Genomic DNA was extracted using an Aidlab extraction kit (Aidlab, Beijing, China) following the manufacturer’s protocol. Paired-end (PE) read libraries with an insert size of 350 bp were constructed in accordance with the Illumina library preparation protocol, followed by sequencing on an Illumina HiSeq 2000 platform (Illumina, San Diego, CA, USA). Target sequencing coverage for all samples was 25× (Table S1).

Reads with quality scores lower than 20 were removed, as well as adapter sequences using Trimmomatic (v0.36) [41]. Reads shorter than 36 bases after the above trimming were excluded from further analyses. Due to the high-quality assembly and annotation, as well as highly conserved homology between *P. davidiana* and *P. trichocarpa* genomes [42,43,44], our filtered reads were mapped to the *P. trichocarpa* reference genome (v3.0) [42] using BWA-MEM in bwa-0.7.15 [45] using default settings. Next, sorted bam files were generated from sam files using SortSam in Picard (v1.96). Reads with identical external coordinates and insert lengths were removed to prevent PCR duplication using MarkDuplicates in the Picard toolkit (http://broadinstitute.github.io/picard/, accessed on 18 June 2020). After raw data filtering, sequence alignment, sequence sorting, and PCR repeat removal, only the reads with the highest base quality were employed for downstream analyses.

### 2.2. Site Filtering and SNP Calling

For bam files, sites were removed that had (1) reads that had multiple best hits, (2) a flag above 255, (3) a minIndDepth of less than 10 individuals, and (4) a mapQ of less than 50 for one individual; (5) only proper pairs (pairs of reads with both mates mapped correctly) were retained. Nine samples (three from each sampling area) were selected from the north, central, and southwest populations to obtain the depth of reads using SAMtools, with 500,000 rows randomly selected to create the distribution map (Figure S4). Population genetic statistics obtained by the inferred site-frequency spectrum (SFS) were estimated directly in ANGSD (v0.921) [46] rather than by calling the genotype. We assumed the state of the *P. trichocarpa* reference genome as the ASB. The site allele frequency likelihood was calculated based on the SAMtools [47] genotype likelihood model for all sites using doSaf, and a maximum likelihood estimate of the expanded SFS was obtained through the expectation maximization (EM) [48] algorithm using realSFS. Several population genetic statistics were then calculated according to the global SFS.

Multi-sample SNP and genotype calls were implemented by HaplotypeCaller and GenotypeGVCFs in GATK v3.7 [49]. We performed several filtering steps to prevent false positives in SNP and genotype calling: (1) sites with extremely low (<10 reads for each individual) or high (>150 reads for each individual) read numbers were removed after inspection; (2) SNPs with more than two alleles in the three populations were removed; (3) SNPs at or within 5 bp from any indels were removed; (4) SNPs within 10 bp were all removed if two or more existed; (5) genotype quality (GQ) scores <10, mapping quality (MQ) <40.0, quality by depth (QD) <2.0, and depth (DP) <10.0 were classified as missing genotypes, and therefrom SNPs with more than two genotypes missing in each population were excluded.

### 2.3. Population Structure

NGSadmix [50], a part of the ANGSD package [46], was employed to infer population genetic structure using only sites that contained less than 10% missing data. In order to estimate genotype likelihoods, we used ANGSD (v0.921) with the SAMTools model [47]. Then, a Beagle file was generated for the genome subset, which was regarded as a variable based on a likelihood ratio test (*p*-value < 10^−6^) [48]. The number of genetic clusters K was tested from 2 to 10, the maximum number of iterations based on the EM algorithm was set to 10,000, and the error rate at each K value was calculated using ADMIXTURE (v1.3.0) [51]. 

A principal component analysis (PCA) was conducted to visualize interindividual genetic relationships using PLINK (v1.90b5) [52], which considered sequencing errors and uncertainty in genotype calls [53]. Two types of files (eigenval file and eigenvec file) were then obtained, in which the eigenval file represents the proportion of each PCA, and another file recording feature vector was used to draw the PCA results.

### 2.4. Demographic Modelling

In order to provide additional insight into the observed population structure, different demographic models were run and compared to see which were most plausible in generating the currently observed genetic structure. Alternative demographic models were compared, and demographic parameters were inferred using a coalescent simulation-based method implemented in *fastsimcoal2.6* (v2.6.0.3-14.10.17) [54]. A two-dimensional joint SFS (2D-SFS) was constructed based on the posterior probabilities for sample allele frequencies using ngsTools. Corresponding to the analyses of NGSadmix and PCA, a total of 21 models with different divergence and introgression scenarios were simulated (Figure S1). First, four models without post-differentiation gene flow were simulated. Of the four models, three first subdivided the ancestral population into two groups, involving the bifurcating topologies of all likely assumptions for the three populations. The fourth model directly divided the ancestral population into three groups, reflecting a simultaneous divergence of all three populations at a single point in time (hard polytomy). Then, based on the basic model determined in the above steps, different scenarios of *N*_e_ were simulated before and after differentiation, from which a better fitting model was identified for each population. Finally, the occurrence of gene flow was considered after differentiation in this model. In each model, the setting ranges for the parameter estimation are shown in Table S2a, and the expected 2D-SFS (Table S2b) and log-likelihood for a set of demographic parameters were estimated using 100,000 coalescent simulations. We set 40 conditional maximum algorithm cycles in each run and obtained global maximum likelihood estimates for each model from 50 independent runs. The maximum likelihood value of 50 independent runs was used to represent the model for comparison using the Akaike information criterion (AIC) and Akaike’s weight of evidence tests [54]. The model with the largest Akaike’s weight value was regarded as the optimal model. The parameter confidence intervals (CIs) for the optimal model were obtained from 100 parametric bootstrap samples, which were run independently 50 times in each bootstrap. When converting estimates to units for years and individuals, we assumed that the mutation rate and the average generation interval time in *Populus* were 3.75 × 10^−8^ per site per generation and 15 years per generation (average time from seed germination to seed production) [55], respectively.

### 2.5. Genome-Wide Patterns of Differentiation

Patterns of genome differentiation between the north, central, and southwest populations were characterized by dividing the genomic data into 40,995 non-overlapping 10-kbp windows. At least 1000 bases after the filtering steps above were required for a window to be included in downstream analysis. Windows with fewer than 10 variable sites were discarded. Then, the degree of genetic differentiation between pairs of the three populations was estimated by calculating *F*_ST_ using VCFtools software (v0.1.13) [49].

### 2.6. Identification of Outlier Regions and Signatures of Selection

In order to examine the effect of natural selection on outlier windows, we performed coalescent simulations using the msms (v3.2rc) [56] program to compare the observed patterns of genetic differentiation (based on *F*_ST_) and those expected for different populations based on the best-fit model simulated by *fastsimcoal2.6*. Corresponding to 10-kbp windows of the same sample size for each population, using the msToGlf program in ANGSD [46] that simulated the genotype likelihood and set a sequencing depth of 27× (the same as the actual average depth) and an error rate of 0.5%, we performed 40,995 replicates of the genotypes (the same number of windows as the actual *F*_ST_ windows). The conditional probabilities (*p*-values) of more extreme *F*_ST_ values in the simulated datasets than those observed in the actual datasets were calculated to assess whether the observed window deviated significantly from the expectation. Multiple tests were then corrected by using the false discovery rate (FDR) adjustment, and only windows with an FDR below 5% were identified as candidate regions affected by selection, which were divided into highly differentiated regions, while the remaining windows were regarded as background variability.

The highly differentiated and background regions of the genome were compared by means of several population genetic statistics in the three populations. First, nucleotide diversity (θ_π_), Tajima’s *D* [57], and Fay and Wu’s *H* [58] were calculated based on the sample allele frequency likelihoods of non-overlapping 10-kbp windows in ANGSD (v0.921) [46]. Next, linkage disequilibrium (LD) and recombination rates across populations were evaluated. The assessment method for LD in each 10-kbp window was used to calculate the correlation coefficient (*r*^2^) between SNPs with a distance of more than 1 kbp by VCFtools [49]. We used LDhat 2.2 [59] to estimate the recombination rates (ρ) across populations, with 1,000,000 Markov chain Monte Carlo (MCMC) iterations, sampling every 2000 iterations, and a block penalty parameter of five. The absolute measure of divergence (d_xy_) was calculated by the posterior probability of sample allele frequency per site using the ngsStat [53] program and averaged over each 10-kbp window between populations. 

### 2.7. Proportion Calculations of ASB and DB

The proportions of ASBs and DBs fixed in the whole genome were calculated. Assuming the reference genome *P. trichocarpa* to be the ancestral state, if the base is the same as the ancestor, we refer to it as the “ancestral allele”; if it is different from the ancestral state, we refer to it as a “derived allele”, calculating the “derived allele” frequency (daf) using ANGSD (v0.921) [46] to create a DAF file. Based on the DAF file, we wrote an R script counting the proportion of shared nucleotide diversity, fixed ASBs, fixed DBs, and fixed differences for each window, the standards for the last three calculations are as follows: for each population, the maximum and minimum range of “daf” were set to “high 1” and “low 0”. For example, in both the central (C) and southwest (SW) populations:

Fixed ASB: SW_daf_ = 0 and 0 < C_daf_ < 1. An ASB represents that the offspring population have the same base state as the ancestral population at the same site. In our study, we assumed that the reference genome (*P. trichocarpa*) at a certain locus is “A”: (1) this locus is polymorphic in the central population and one of these bases is “A”, thus the “daf” in the central population is 0 < C_daf_ < 1. Meanwhile, (2) all individuals of the southwest population have fixed the “A” base at this locus, that is, the SW_daf_ = 0 in the southwest population. If the above two points are included, we considered that ASBs are fixed in the southwestern population, but in the central population have not yet been fixed, so we classified this scenario as a fixed ASB in the southwest population. We used the same method to calculate the proportion of fixed ancestral adaptive bases in other populations. 

Fixed DB: SW_daf_ = 1 and C_daf_ < 1. A DB represents that the offspring population have a base state different from the ancestor at the same site when adapting to the new environment. First, we assumed that the reference genome (*P. trichocarpa*) at a certain locus is the “A” base, but all individuals of the southwest population are the “T” base, different from the reference genome, that is, “A” does not exist in the southwest population, the “daf” in the southwest population is SW_daf_ = 1, but the central population may exist as “T” base (not fixed) or may not exist as this base, so C_daf_ < 1. That is, a DB has occurred and has been fixed in the southwest population but has not yet been fixed in the central population, so we classified this scenario as a fixed DB in the southwest population. We used the same method to calculate the proportion of fixed new mutated bases in other populations. 

Then, we calculated the fixed proportion of ASBs and DBs in the total base in each population, and the fixed proportion of ASBs to DBs. The Mann–Whitney U test was employed to determine significant differences in all of the above-mentioned population genetic statistics between highly differentiated windows and background windows.

### 2.8. Gene Identification and Sequence Analysis in Highly Differentiated Regions

Based on the genome feature file (.gff) of *P. trichocarpa*, we identified and annotated genes in the highly differentiated regions of the north-central populations and central-southwest populations, respectively. Interestingly, the *REF6* gene is associated with “flowering”, which may be related to the normal survival and reproduction of plants in the Yunnan–Guizhou plateau region; thus, we selected coding DNA sequences (CDSs) from *REF6* for further analyses. Molecular diversity indices including segregating sites (S), nucleotide diversity parameters (π), Watterson’s θ_w_ [60], and the number of haplotypes were calculated for each locus. Tajima’s *D* [57] and Fu and Li’s *D* and *F* [61] were also estimated to determine the extent of consistency of the data with neutral evolutionary models, using DnaSP (v6.12.03) [62]. With *P. trichocarpa* as an outgroup, distance trees for each CDS DNA haplotype were constructed in MEGA5 (v5.05) using the neighbor-joining method. In order to ensure sufficient variability and to show that the network map was not too complex, one CDS with suitable numbers of variant loci and haplotypes was used to construct a haplotype network in Network (v10.2) using the median joining method [63] to infer relatedness between individuals.

## 3. Results

We obtained whole-genome sequence data of 90 *P. davidiana* distributed in three geographic regions of China, and the clean sequencing reads were mapped to the *P. trichocarpa* reference genome (v3.0) [42], with an average mapping rate of 84.29% of filtered reads (Table S1). In the north, central, and southwest populations, the average coverage of reads mapped uniquely per site for samples was 27.12, 28.74, and 26.89 (Table S1), respectively, and a total of 3,452,133 SNPs, 3,470,296, and 3,598,404 high-quality SNPs were identified, respectively.

### 3.1. Population Structure

The individual ancestry and genetic structure of the different populations were inferred from genotype likelihoods. The number of genetic clusters (K) was set from 2 to 10, with the lowest minimum K-value error rate for K = 3 (cross-validation error = 0.280) as calculated in ADMIXTURE (v1.3.0). All sampled individuals were divided clearly into population-specific genetic clusters. At K = 2, the southwest individuals clustered together, and the northern and central individuals all clustered together with little to no cross assignment (Figure 1d). When K = 3, individuals from the north and central populations subdivided but with some evidence of admixture between these populations, while individuals in the southwest group remained separate. At K of 4 and 5, internal subdivisions within the southwestern and central populations were noted, but no meaningful admixture between the southwestern genetic clusters and the other two was inferred. The PCA results recapitulated the NGSadmix results with individuals from each of the three collection sites clustering together and separate from individuals collected at other sites (Figure 1c).

### 3.2. Divergence and Demographic Reconstructions

**Figure 2:**
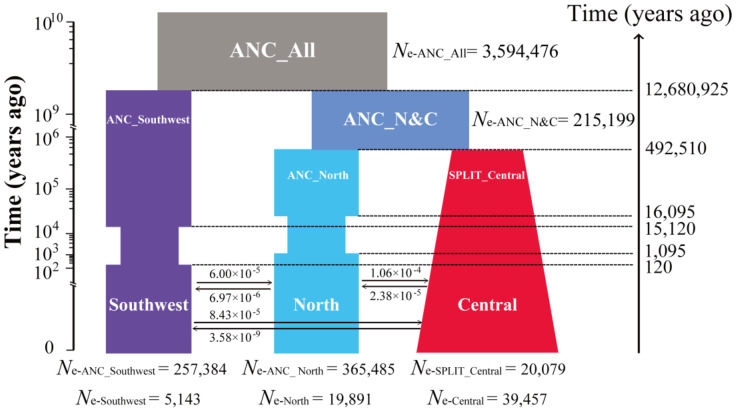
Demographic history of *P. davidiana*. A simplified graphic for the best supported model inferred by *fastsimcoal2.6*. The *N*_e_ represents the effective population size. The ancestral population (ANC_All) of three populations is colored gray, the ancestral population of the north and central populations (ANC_N&C) is colored gray-blue, and the north, central, and southwest populations are colored blue, red, and purple, respectively. The widths represent the relative *N*_e_. Double-headed arrows represent the per-generation gene flow between pairs of the three populations. All estimations of demographic parameters are shown in Table 1. The neutral mutation rate of each generation (µ) and the generation time was 3.75 × 10^−8^ per site per generation and 15 years in *Populus*, respectively.

The demographic history of the three *P. davidiana* genetic clusters was inferred using *fastsimcoal2.6* based on a continuous-time coalescent simulation. Summary statistics and the relative likelihood for 21 demographic models associated with Figure S1 are shown in Table S2c,d, respectively. The best-fitting model was an isolation-with-migration model (Figure 2), and the exact parameter estimates with divergence time, gene flow, and *N*_e_, as well as the estimates of their associated 95% CIs, are provided in Table 1. The best-fitting model indicated that the southwestern lineage first split from an ancestral population approximately 12.68 million years ago (Mya) (bootstrap range [BR]: 4.32–14.78 Mya) and did not infer gene flow during the early stages of differentiation between these lineages. The north and central lineages were inferred to have diverged approximately 0.49 Mya (BR: 0.21–0.68 Mya). Bottlenecks were inferred to have occurred in the southwest and northern lineage approximately 15,120 years ago (ybp) (BR: 15,030–16,629 ybp) and 16,095 ybp (BR: 15,097–52,751 ybp), respectively. The bottlenecks in the southwest and north lineages were inferred to had recovered very recently at 120 ybp (BR: 30–1629 ybp) and 1095 ybp (BR: 97–37,751 ybp), respectively. The *N*_e_ at different periods for each population and the estimated migration rates per generation (m) between lineages varied by several orders of magnitude with rates between the southwest and other two lineages being the lowest (Figure 2; Table 1).

### 3.3. Genome-Wide Patterns of Differentiation and Identification of Outlier Regions

Patterns of inter-population genetic differentiation across the genome were calculated through analysis of *F*_ST_-based relative divergence in nonoverlapping 10-kbp windows. When comparing windows between the southwest population and either of the other two populations, a relatively high mean *F*_ST_ was calculated (mean *F*_ST_ = 0.2331 ± 0.1377 between southwestern and northern; mean *F*_ST_ = 0.2066 ± 0.1372 between southwestern and central), whereas the mean *F*_ST_ (0.0778 ± 0.0587) between the central and northern populations was much smaller—indicating less divergence on average across the entire genome (Figure 1b; Table S3). 

**Figure 3:**
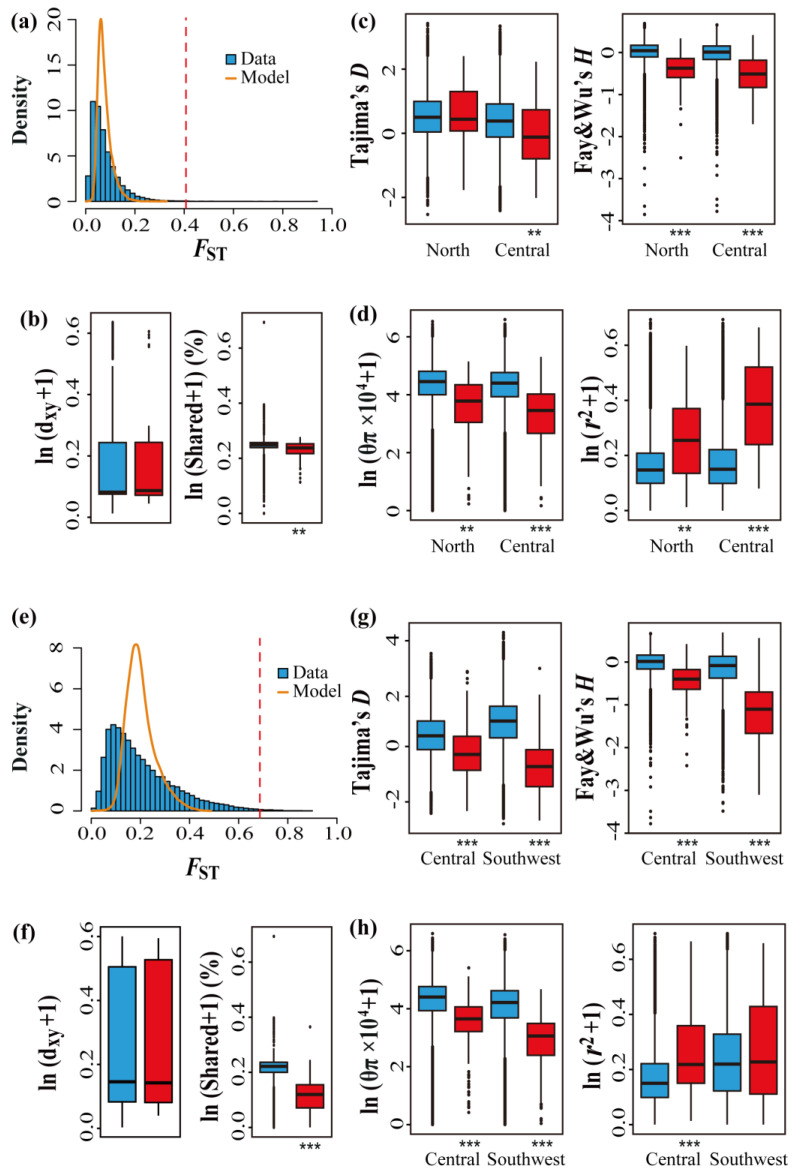
Identification of candidate outlier windows that may be affected by natural selection. (**a**–**d**) Genetic parameters between the north and central populations. (**e**–**h**) Genetic parameters between the central and southwest populations. (**a**,**e**) Distribution of genetic differentiation (*F*_ST_) between two populations from the observed (blue bar) and simulated datasets (orange line). The dotted line represents the thresholds for determining significantly (false discovery rate <5%) high (red bars) genetic differentiation based on coalescent simulations. (**b**,**f**) Comparisons of d_xy_ (absolute measure of divergence) and the proportion of inter-population shared nucleotide diversity between regions with significantly high genetic differentiation (red boxes) and the genomic background (blue boxes). (**c**,**g**) Comparisons of Tajima’s *D* and Fay and Wu’s *H* between regions with significantly high genetic differentiation (red boxes) and the genomic background (blue boxes). (**d**,**h**) Comparisons of nucleotide diversity (θ_π_) and LD (*r*^2^) between regions with significantly high genetic differentiation (red boxes) and the genomic background (blue boxes). Asterisks indicate significant differences between high-genetic-differentiation and background genomic regions based on Mann–Whitney U tests (** *p*-value < 1 × 10^−4^; *** *p*-value < 2.2 × 10^−16^).

The datasets simulated using msms (v3.2rc) [56] showed that the means of 40,995 *F*_ST_ replicates were 0.0761 ± 0.0278 (north and central) and 0.2018 ± 0.0593 (central and southwest), respectively, which were very close to the observed *F*_ST_ mean (Table S3). A total of 82 (north and central) and 213 (central and southwest) windows (right tail, FDR < 0.05) were considered to be affected by natural selection by comparing the observed *F*_ST_ datasets with the simulated datasets based on the coalescent simulation, which were divided into highly differentiated regions (Figure 3a,e). The distribution of highly differentiated genomic regions (based on *F*_ST_) in the chromosomes is shown in Figure S2, and the size of the outlier regions was most of 10-kbp (Figure S3).

For the comparison between the central and southwest genetic clusters, the proportion of shared nucleotide diversity in genomic regions of high differentiation was significantly lower compared to the background regions, but there was no significant difference in d_xy_ (Figure 3f; Table S4). Tajima’s *D* values and Fay and Wu’s *H* were negative in highly differentiated regions, and from a Mann–Whitney U test, the values of these two genetic parameters in highly differentiated regions were significantly lower than those in the background regions (Figure 3g; Table S4). In addition, the levels of nucleotide diversity (θ_π_) decreased significantly in highly differentiated regions in both populations, and *r*^2^ between SNPs increased significantly in highly differentiated regions in the central population; however, in the southwest population these differences between high-differentiation and background regions were insignificant because the values in both regions were high and higher than in other populations (Figure 3h; Table S4).

For comparisons between the central and northern populations, most parameters showed a similar trend as those in the central and southwest populations, except Tajima’s *D*, which in the north population showed no significant difference in highly differentiated regions compared to background regions (Figure 3c; Table S4). In summary, the genetic parameters between the highly differentiated region and the background regions were more significantly different in the southwest population compared with the north and central population.

We also found a significant negative correlation between the relative measure of divergence (*F*_ST_) and the recombination rate (ρ), while the absolute divergence (d_xy_) had a less pronounced correlation with the recombination rate (ρ) in the whole genome of each population (Table 2).

### 3.4. Contribution of ASBs and DBs

**Figure 4:**
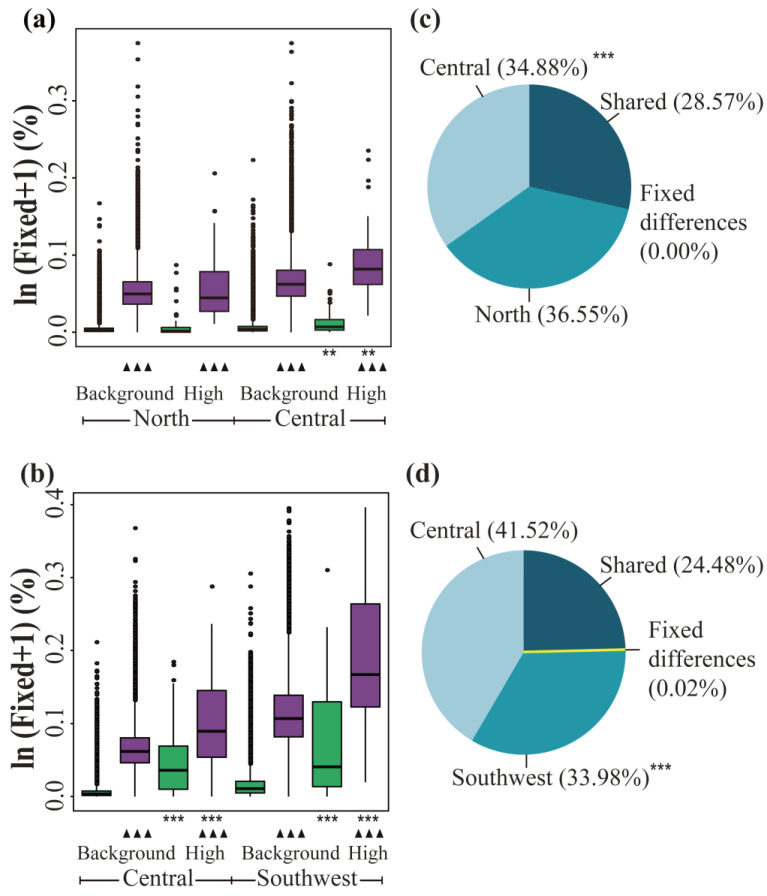
Proportions of fixed alleles, fixed differences, shared, and private nucleotide diversity. (**a**,**b**) Comparisons of fixed alleles between regions with high genetic differentiation and the genomic background. Green boxes represent the proportion of fixed derived alleles arising from derived bases (DBs) in the north and central populations, and in the central and southwest populations; purple boxes represent the proportion of fixed ancestral-state bases (ASBs) in the north and central populations, and in the central and southwest populations. Asterisks indicate significant differences between high-genetic-differentiation and background genomic regions, and triangles indicate significant differences between ASBs and DBs, based on Mann–Whitney U tests (** *p*-value < 10^−4^; ***/^▲▲▲^ *p*-value < 2.2 × 10^−16^). (**c**,**d**) The pie chart shows the proportion of fixed differences and shared and private nucleotide diversity in the north and central populations, and in the central and southwest populations. Asterisks indicate significant differences between the private gene proportions of the two populations, based on Mann–Whitney U tests (***^/▲▲▲^ *p*-value < 2.2 × 10^−16^).

From the perspective of highly differentiated regions and background regions, besides the ASBs in the north population, the proportions of fixed bases in highly differentiated regions were greater than the background averages, not only for DBs but also for ASBs (Figure 4a,b; Table S4). From the perspective of the different sources of variation, the Mann–Whitney U test showed that the fixed ASBs were significantly more abundant than DBs in highly differentiated regions and background regions of each population (Figure 4a,b; Tables S3 and S4). The contribution of ASBs relative to DBs is shown in Table 3: for the whole genome, the fixation of ASBs was 13.19–19.03 times that of DBs (Figure S5); for the background regions, the proportions were 13.22 to 19.03; and the high-differentiation regions were 4.64 to 13.15. Compared to the proportions in the background regions, the proportions in the highly differentiated regions all showed a decreasing trend, indicating that the number of DBs in the high-differentiation regions increased significantly. The southwestern population was most prominently characterized, as the fixed values of ASBs and DBs were the highest in highly differentiated regions.

Of the total number of polymorphisms between the north and central populations and the central and southwest populations, fixed differences accounted for 0.00% and 0.02%, respectively, whereas 28.57% and 24.48% of polymorphisms were shared between them, respectively, with the remaining polymorphic sites made up of private alleles (Figure 4c,d). In contrast, the southwest population had the lowest proportion of private alleles among the three populations.

### 3.5. Genes under Selection

After gene annotation with the *P. trichocarpa* genome as the reference, a total of 59 (north and central) and 175 (central and southwest) genes were identified in outlier windows that showed the highest levels of differentiation based on *F*_ST_ values in the respective datasets (Tables S5 and S6). The *REF6* gene was identified in the central and southwest populations, which may be related to the early flowering of alpine plants to adapt to the environment. Eight CDSs were present in *REF6*. The neutrality tests (Tajima’s *D*, Fu and Li’s *D* and *F*) (Table 4) for *REF6* CDSs were significantly negative in the southwest population; such results are indicative of strong positive selection on these loci within the southwest population. The nucleotide diversity measure and S for the CDSs from *REF6* showed a similar pattern, which further supported the conclusion that the southwest genetic cluster has undergone strong positive selection for these altered gene variants.

**Figure 5:**
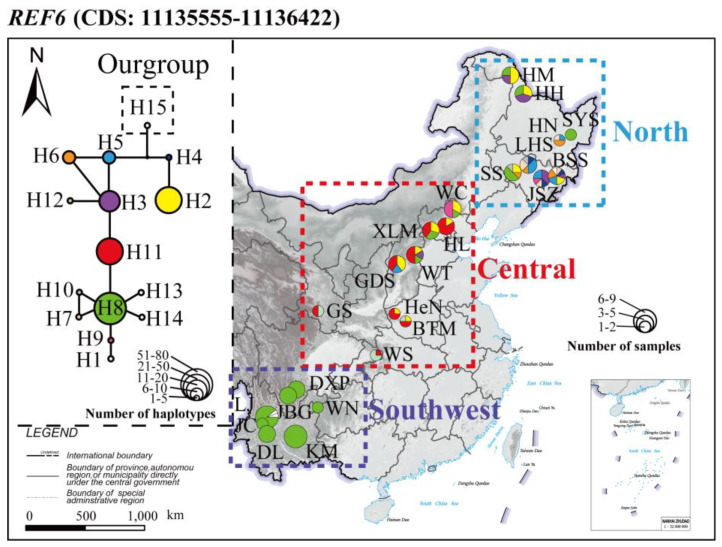
Frequencies and relation of DNA haplotypes of the *REF6* gene across the population range of *P. davidiana*. One CDS region of *REF6* (11135555–11136422) was selected as the representative haplotype network, and the distribution of haplotypes was marked on a relief map of China. Colored haplotypes are shared by two or more populations of sampling locations, and private haplotypes are not colored. The outgroup is framed in a square. The sizes of circles in the network are proportional to the observed number of individuals in the haplotypes, and the sizes of the circles on the map are proportional to the population sizes of sampling locations.

A distance tree of each CDS from *REF6* from each individual from all the populations was constructed with *P. trichocarpa* as an outgroup to assess the degree of clustering and branching order for each CDS across the entire sample set (Figure S6). The CDS partitions showed different tree topologies with southwest population terminals often clustered in derived positions of the tree relative to the outgroup. The geographical distribution of the haplotype network for the *REF6* gene (CDS: 11135555–11136422, with suitable numbers of variant loci and haplotypes) is displayed on a relief map of China (Figure 5), with fifteen haplotypes. Three haplotypes were common (frequency >10%): H8 (43.33%), H11 (15.56%), H2 (13.89%). For each population, H8, as the most common haplotype in the north population and southwest population, was represented by 21.67% and 96.77% between the two populations, respectively. H11 (48.28%) was the most common in the central population.

## 4. Discussion

Consistent with previous findings [36,40], NGSadmix and PCA results supported the division of *P. davidiana* into three groups; our results further confirm the conclusions of Zheng et al. [40] and Hou et al. [36] that *P. davidiana* in the southwest region is severely differentiated compared to other regions (Figure 1, Figure 3, Figure 4 and Figure S2b) and that significant gene flow can be detected between the southwest and other regions (Figure 2, Table 1). Our work herein provides three important components to previous work on the *P. davidiana* species and the components are discussed below in separate sections.

### 4.1. Reconstruction of Historical Demography as Relates to East Asian Geology and Climate Fluctuations

Evolution can be strongly influenced through abiotic processes such as mountain uplift and associated climatic fluctuations [64,65,66]. Our simulation-based analyses indicated that the southwest population began to differentiate from the ancestral population approximately 12–13 Mya. The divergence of *Populus* lineages in this part of Asia could have been induced by formation of the high central plateau of the Qinghai–Tibet Plateau (QTP) during the Neogene [67], which was well under way by 10–13 Mya [68]. The uplift of the QTP had an important influence on the local climate of Asia, as well as possible worldwide impacts [69,70,71,72,73], including the Yunnan–Guizhou plateau [74]. The divergence between the southwest lineage and north-central lineages was nearly congruent with the estimation of Yang et al. [74] that the two major clades (*Corylus yunnanensis* and *C. heterophylla-C. kweichowensis*) occurred separately at approximately 12.89 Mya, which reflects the environmental particularity of the Yunnan–Guizhou Plateau and the reliability of the divergence event. Such orogenic changes are likely to have resulted in a change in the selective landscape and could have favored certain alleles evolving in these parts of the ancestral species range.

Divergence occurred between the north and central populations approximately 0.5 Mya, corresponding to the Middle Pleistocene (0.13–0.78 Mya), a period of climatic and environmental change, during which the expansion of ice caps had significant effects on plant species ranges in the northern hemisphere [75,76]. Evidence for this can be found in pollen cores from the area around Beijing (China), wherein decreases in pine and deciduous trees pollens corresponded to increased winter monsoons in East Asia around 0.5 Mya [77], which is consistent with the decreases in *N*_e_ inferred in the southwest and north populations around the same time (Figure 2). The continuous growth among the central population might be related to this being the site of glacial refugia and the bottlenecks in the north and southwest populations being related to stronger constrictions in the refugial areas for these lineages [78,79].

### 4.2. ASBs and DBs in the Adaptation to Changing Selective Pressures

When selection is one of the dominant evolutionary forces affecting patterns of genetic differentiation among species, genomic regions with low recombination are expected to present increased *F*_ST_ values without changes in d_xy_ values [9,80]. In highly differentiated genomic regions of *P. davidiana*, a significant negative correlation between the *F*_ST_ and ρ, and a less pronounced correlation between d_xy_ and ρ (Table 2), highlighted the important role of linked selection [81], which is consistent with the findings of Wang et al. [6]. The assessment of multiple population genetic parameters further supported our findings (Figure 3; Table S4). Compared with the background regions, the highly differentiated regions showed that Tajima’s *D* and Fay and Wu’s *H* tended to have more negative values, hence suggesting strong natural selection [82]. Reduced nucleotide diversity (θ_π_), lower proportions of shared nucleotide diversity in the highly differentiated genomic regions, and higher *r*^2^ values (the northern and central populations were higher in highly differentiated regions than background regions, and the southwest populations had high values in both regions) [83] further revealed selective signatures.

The most frequent source of adaptive alleles is from ancestors, as has been shown in numerous population genetics studies [13,14,19,84], as well as the results herein; ASBs exceeded DBs and thus played the most important role in species adaptation (Figure 4a,b; Table S4). For the different genomic regions of *P. davidiana* populations, all the proportions of two sources of genetic variation in the highly differentiated regions were significantly higher than those in the background (Figure 4a,b; Table S4); and the proportion of ASBs to DBs was significantly lower in the highly differentiated regions than the background (Table 3), which is indicative of a significantly increased proportion of the DBs, especially in the southwest population. This pattern supported our hypothesis that the proportions of DBs in the genomic regions with strong natural selection increased significantly to adapt to the extreme environment pressures which ASBs are insufficient to cope with. For the southwest population strongly affected by natural selection (compared to the other two populations), the ASBs and DBs across the whole genome were highest (Table S3), respectively, which may be due to the geographical distribution regions of southwest population which are (mainly distributed in southern Sichuan Province, western Guizhou Province, and Yunnan Province, China) markedly different in climatic factors such as temperature, humidity, light intensity, and day length than those experienced by northern populations.

A bottleneck effect occurred after the southwestern population split from the ancestral population. If the bottleneck effect plays a major role compared to natural selection, a neutral test such as Tajima’s *D* and Fay and Wu’s *H* will not be able to detect significant negative values in the highly differentiated regions of the southwest population (our results reject this hypothesis). The trend of the bottleneck effect affecting the DBs and the ASBs is similar, that is, if the bottleneck effect causes the frequency of DBs to increase and become fixed (or lead to reduced frequency or even loss), the bottleneck effect would also promote the ASB to be selected and fixed (or lead to reduced frequency or even loss), thus both DBs and ASBs will exhibit a similar proportion of reduced diversity due to the bottleneck effect, which will not affect the conclusions of the above study. Besides this, the effect of the hitchhiking influence on polymorphism is ubiquitous during natural selection. When a locus has been fixed due to natural selection, the surrounding variant loci are also fixed due to tight linkage effects. This has the same effect on both DBs and ASBs, which indicates the fixed sites of DBs and ASBs within the neutral site region would increase in the same proportions; however, this effect is limited [85], thus the proportion of ASBs to DBs (Table 3) will not be significantly affected in our results. Similarly, the hitchhiking effect is identical across the whole genome and thus insufficient to impact conclusions on highly differentiated and background regions regarding differences in the proportion of ASBs and DBs.

Significantly, however, ASBs can come from multiple sources. As has been mentioned, introgression is one such source and has been documented among numerous lineages in *Populus*. If introgression was a recent source of ASBs for the southwest population from other species, it would result in increases in recombination rates and decreases in measures of divergence [86]. The recombination rate (ρ) in the southwest population was 15.0683% versus 17.8010% in the central and 18.5441% in the northern population. Similarly, *F*_ST_ and d_xy_ were higher between the southwest and the central and northern populations than they were between the central and northern populations (Table S3). Lastly, if recent introgression had occurred between the southwestern and other species, this would be detectable in increased private alleles and decreased nucleotide diversity. The above results were mutually exclusive with the hybridization scenario (Figure 4c,d; Table S3). However, further comparisons should also be conducted with species which are closely related to the *P. davidiana* to assess whether introgression with these species could have increased ASBs in the southwestern population of *P. davidiana*.

Notably, assuming that the reference genome is an ancestral state is not an absolutely safe assumption, the reference genome may carry the very few derived allele sites that are variable. Future studies could be based on the ancestral states for multiple nodes in the *Populus* phylogeny to check how incorrect ancestral-state specification affects inferences, which can improve the preliminary results of our study on the quantitative study of ASBs and DBs. Overall, the adaptive evolution of *P. davidiana* in the southwestern portion of the range is a representative example in which a combination of ASBs and DBs were both utilized in the genomic evolution of this population. This study provides a useful comparative dataset for similar studies of adaptive evolution in *Populus* specifically and trees more generally.

### 4.3. Genes Related to Environmental Adaptation

*P. davidiana* is widely distributed in East Asia with a large latitudinal span, the temperature ranges from that of a cold temperate zone to that of a subtropical zone, with great climatic changes and significant altitudinal differences. As a result of high environmental differences in the southwest region relative to the north and central regions of China, several genes that might experience strong selection to respond to a high-altitude subtropical climate were identified in *P. davidiana* (Table S6). Our data show that certain highly divergent regions of the *P. davidiana* genome are under strong selection in different parts of the species range; this is also true at the *REF6* CDS level with different levels of sequence conservation found among different CDSs and in different populations. This is also evident in the haplotype networks with a higher abundance of a single haplotype in the southwestern population and a more even distribution of haplotypes in the central and northern populations (Figure 5), just as the proportion of the H8 haplotype in the southwest population was much higher than that in the northern and central populations, which may be under positive selection in adapting to the southwest region. The neutral test index of Tajima’s *D* and Fu and Li’s *D* and *F* significantly deviated from the neutral model in the southwest population, and the values of reduced nucleotide polymorphisms in the southwest population compared to the northern and central populations also indicated that the southwest population experienced natural selection and that local adaptation had occurred to adapt to the Yunnan–Guizhou Plateau.

**Figure 6:**
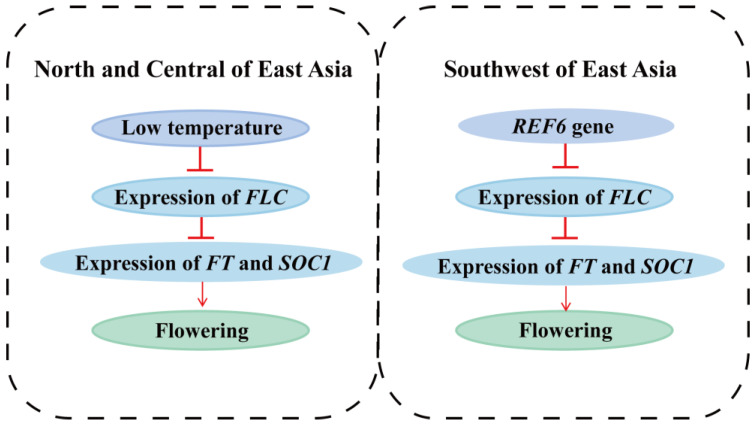
*REF6* gene controlling flowering time. Inhibitory action is represented by a horizontal line under a vertical line, promoting action is represented by an arrow of a solid line. *FT* and *SOC1* promote flowering, but both are inhibited by *FLC*. In northern and central East Asia, the expression of *FLC* in *P. davidiana* is inhibited in winter because of low temperatures and thus controls flowering time, while the temperature in southwestern East Asia is not sufficiently low to inhibit *FLC* expression. Expression of the *REF6* gene has an important inhibitory effect on the *FLC* gene, and thus plays a pivotal role in promoting flowering.

The *REF6* gene is closely associated with *FLC* gene. *FLC*, as a major repressor of flowering, plays a pivotal regulatory role in the vernalization pathway. Sheldon et al. [87,88] reported that low temperature (vernalization) served as negative regulator of *FLC* mRNA and protein levels: the longer the treatment time was, the weaker the *FLC* expression, thus promoting flowering. They also found that late-flower ecotypes and overexpression mutants had high *FLC* expression levels, while early-flower ecotypes and non-functional mutants had little or no activity [87,88]. Overall, the stronger the *FLC* expression, the later the flowering. *REF6* is an *FLC* repressor: loss-of-function mutations in *REF6* lead to increased expression of the flowering repressor *FLC* and hence late flowering, and overexpression of *REF6* causes increases in *FT* and *SOC1* mRNA levels in an *FLC*-independent manner that leads to the early-flowering phenotype [89]. In winter, the expression of *FLC* in the *P. davidiana* north population is inhibited due to low temperatures, which leads to appropriate flowering conditions, while such temperature cues are not present in the southwestern portion of the species range. The *REF6* gene is known to affect the regulation of flowering time by inhibiting the expression of *FLC* (Figure 6) [87]. Thus, the *REF6* may play a pivotal role in promoting flowering to adapt to the climate in the southwestern populations of *P. davidiana*. This is our speculation and needs to be verified by further experiments in the future.

## 5. Conclusions

Our study provides insights into the evolutionary history of adaptation to new environments by different lineages of *P. davidiana* in East Asia. In particular, the contribution of ASBs relative to DBs in adaptive evolution was quantified, and we found that ASBs exceed DBs but that DBs are important in adapting to new environments. The uplift of the QTP and climate transformation in the Middle Pleistocene may have driven the initial differentiation of *P. davidiana,* as the estimated dates for divergence align with these geological events. Later divisions and bottlenecks may have been associated with more recent events such glaciation. Multiple population genetic parameters demonstrated that linked selection played a pivotal role in genome differentiation. Several genes were identified as being strongly associated with adaptation to the unique climactic conditions present in the southwestern portion of the species range of *P. davidiana*, such as *REF6*.

## Figures and Tables

**Table 1:** Demographic parameter estimates of the best model in Figure 2.

	Point Estimation	95% CI ^a^	
Parameters		Lower Bound	Upper Bound
N_e−ANCAll_	3,594,476	152,843	4,630,207
N_e−ANC−N&C_	215,199	62,862	1,454,818
N_e−ANC_Southwest_	257,384	135,586	461,135
N_e−ANC_North_	365,485	105,719	453,562
N_e−SPLIT_Central_	20,079	8815	29,231
N_e−BOT−Southwest_	1065	916	1164
N_e−BOT−North_	3698	3468	8946
N_e−North_	19,891	5271	54,619
N_e−Central_	39,457	28,082	57,748
N_e−Southwest_	5143	5082	10,794
MIG_Central→Southwest_	3.58 × 10^−9^	2.62 × 10^−11^	2.97 × 10^−6^
MIG_Southwest→Central_	8.43 × 10^−5^	3.00 × 10^−5^	2.40 × 10^−4^
MIG_Central→North_	2.38 × 10^−5^	5.25 × 10^−6^	4.68 × 10^−5^
MIG_North→Central_	1.06 × 10^−4^	8.81 × 10^−5^	1.40 × 10^−4^
MIG_Southwest→North_	6.00 × 10^−5^	4.63 × 10^−11^	1.73 × 10^−4^
MIG_North→Southwest_	6.97 × 10^−6^	6.30 × 10^−6^	1.51 × 10^−5^
T_DIV− Southwest _ANC−N&C_	12,680,925	4,323,255	14,781,162
T_DIV−North−Central_	492,510	214,723.80	680,931
T_BOT−Nend−Southwest_	120	30	1629
T_BOT−Nstart−Southwest_	15,120	15,030	16,629
T_BOT−Nend−North_	1095	97	37,751
T_BOT−Nstart−North_	16,095	15,097	52,751
GrowthP_−Central_	−2.06 × 10^−5^	−8.00 × 10^−5^	−4.34 × 10^−6^

Notes: The parameters are defined in Figure 2. *N*_e−North_, *N*_e−Central_, *N*_e−Southwest_, *N*_e−ANCAll_, *N*_e−ANC−N&C_, *N*_e−ANC_Southwest_, *N*_e−ANC_North_, *N*_e−SPLIT_Central_, *N*_e−BOT−Southwest_, and *N*_e−BOT−North_ represent the effective population size of the present north population, present central population, present southwest population, ancestor of the three populations, ancestor of the north and central populations, early split southwest population, early split north population, early split central population, southwest population during the bottleneck period, and north population during the bottleneck period, corresponding to the number of individuals for diploid species. MIG_Central→Southwest_ and MIG_Southwest→Central_ represent the migration rate of each generation from the central population to the southwest population, and that from the southwest population to the central population; migration rates between other populations are represented in the same way. TDIV_−Southwest_ANC−N&C_ and TDIV_−North−Central_ represent the estimated divergence time of the southwest population and north−central populations, north population, and central population. T_BOT−Nend−Southwest_ and T_BOT−Nstart−Southwest_ represent the end and start time of the bottleneck in the southwest population, T_BOT−Nend−North_ and T_BOT−Nstart−North_ represent the end and start time of the bottleneck in the north population. GrowthP_−Central_ represents the rate of expansion of each generation from now to the beginning of the division in the central population, obtained from *fastsimcoal2.6*. ^a^ Parameter bootstrap estimation obtained by performing parameter estimation from 100 simulated datasets based on the total maximum composite likelihood estimates displayed in the point estimation column; per likelihood is estimated from 100,000 simulations.

**Table 2:** Spearman’s rank correlation coefficient. Correlation coefficient between the relative measure of divergence (*F*_ST_) and recombination rate (ρ), as well as between absolute divergence (d_xy_) and recombination rate (ρ).

Parameters		Population	Spearman’s ρ	*p*-Value
*F*_ST_ and ρ	North-Central	North	−0.362	<0.01
		Central	−0.337	<0.01
	Central-Southwest	Central	−0.369	<0.01
		Southwest	−0.346	<0.01
d_xy_ and ρ	North-Central	North	0.018	<0.01
		Central	0.016	<0.01
	Central-Southwest	Central	0.012	<0.05
		Southwest	0.027	<0.01

**Table 3:** The mean of the proportion of ASBs to DBs for each window. N-C represents proportions in the north and central populations, C-S represents parameters in the central and southwest populations. The lower quartile and upper quartile are shown in parentheses.

	Population	Highly Differentiated	Background	Whole Genome
N-C	North	13.15 (5.82, 16.30)	19.03 (8.49, 23.75)	19.03 (8.49, 23.74)
	Central	10.04 (4.21, 12.64)	17.90 (7.85, 21.69)	17.89 (7.84, 21.67)
C-S	Central	4.64 (1.69, 4.39)	17.87 (7.46, 22.03)	17.79 (7.40, 21.92)
	Southwest	6.66 (2.02, 8.25)	13.22 (5.64, 15.13)	13.19 (5.61, 15.08)

Notes: The mean of the proportions was obtained by calculating the proportion of ASBs to DBs in each window across the whole genome. ASBs are 4.64–19.03 times higher than DBs, and the reduced proportion of highly differentiated regions is due to the increased number of new mutations (as shown in Tables S3 and S4).

**Table 4:** Nucleotide diversity and neutral test in the CDS region of the *REF6* gene.

Region	CDS	S	π	θ_w_	N_h_	*D*	*D* *	*F* *
North	11135555–11136422	7	0.0027	0.0020	10	0.95	0.55	0.80
	11137806–11137884	0	0.0000	0.0000	1	/	0.00	0.00
	11138254–11138321	1	0.0031	0.0032	2	−0.03	0.53	0.42
	11138432–11138582	0	0.0000	0.0000	1	/	0.00	0.00
	11139034–11139707	7	0.0016	0.0022	9	−0.74	−0.38	−0.59
	11140636–11141367	16	0.0066	0.0082	29	−0.64	0.94	0.43
	11142487–11144538	43	0.0040	0.0045	39	−0.36	1.35	0.85
	11144758–11144891	3	0.0047	0.0048	4	−0.05	0.87	0.69
	Mean	9.63	0.0028	0.0031	11.88	−0.15	0.48	0.33
Central	11135555–11136422	7	0.0021	0.0017	7	0.57	1.23	1.20
	11137806–11137884	1	0.0027	0.0027	2	0.00	0.53	0.44
	11138254–11138321	0	0.0000	0.0000	1	/	0.00	0.00
	11138432–11138582	2	0.0017	0.0029	3	−0.72	−0.93	−1.01
	11139034–11139707	4	0.0014	0.0013	5	0.15	0.99	0.85
	11140636–11141367	14	0.0061	0.0097	26	−1.23	−0.19	−0.68
	11142487–11144538	47	0.0050	0.0055	34	−0.30	−0.28	−0.34
	11144758–11144891	2	0.0035	0.0032	3	0.17	0.73	0.66
	Mean	9.63	0.0028	0.0034	10.13	−0.19	0.26	0.14
Southwest	11135555–11136422	2	0.0001	0.0005	3	−1.44	−2.63 *	−2.64 *
	11137806–11137884	0	0.0000	0.0000	1	/	0.00	0.00
	11138254–11138321	0	0.0000	0.0000	1	/	0.00	0.00
	11138432–11138582	1	0.0006	0.0014	2	−0.71	0.53	0.19
	11139034–11139707	0	0.0000	0.0000	1	/	0.00	0.00
	11140636–11141367	8	0.0117	0.0134	16	−0.42	1.61 *	1.01
	11142487–11144538	9	0.0006	0.0009	11	−1.07	−2.10	−2.08
	11144758–11144891	1	0.0005	0.0016	2	−0.89	0.53	0.13
	Mean	2.63	0.0017	0.0022	4.63	−0.9	−0.26	−0.42

Notes: S: number of segregating sites; π: nucleotide diversity; θ_w_: nucleotide diversity; N_h_: number of haplotypes; *D*: Tajima’s *D* test statistic; *D* *: Fu and Li’s *D* test statistic; *F* *: Fu and Li’s *F* test statistic; * *p* < 0.05.

## Data Availability

The whole-genome re-sequencing data for the *P. davidiana* samples used in this study have been deposited in the China National GeneBank database (CNGBdb) under the project accession number CNP0001249 (http://db.cngb.org/cnsa/, accessed on 8 June 2021).

## Supplementary Materials

The following supporting information can be downloaded at: https://www.mdpi.com/article/10.3390/genes14040821/s1, Figure S1: Tested demographic models; Figure S2: Genome-wide divergence of the chromosome; Figure S3: The physical size distributions (kbp) of regions displaying significantly high genetic differentiation; Figure S4: The distributions of the reads; Figure S5: The proportions of ancestral-state bases/derived bases across the whole genome; Figure S6: Phylogenetic tree of the combined DNA haplotypes; Table S1: Overview information of Illumina re-sequencing data per sample of *P. davidiana*; Table S2: Parameter settings and results for 21 demographic models associated with Figure S1. (a) The setting ranges of the parameter estimation. (b) Two-dimensional joint site-frequency spectrum (2D-SFS) of fastsimcoal demographic inferences. (c) Summary statistic. (d) Relative likelihood; Table S3: Summary population genetic statistics in the whole-genome of *P. davidiana*; Table S4: Summary statistics comparing regions showing high genetic differentiation with background region of *P. davidiana*; Table S5: List of genes located in a region of significantly high genetic differentiation between the north and central populations of *P. davidiana*; Table S6: List of genes located in a region of significantly high genetic differentiation between the central and southwest populations of *P. davidiana*.
