# Supplementary material for: A Genomic Quantitative Study on the Contribution of the Ancestral-State Bases Relative to Derived Bases in the Divergence and Local Adaptation of Populus davidiana
Source: Genes (Basel). 2023 Mar 29;14(4):821. doi: 10.3390/genes14040821 (PMC10137690; doi:10.3390/genes14040821)
Supplement: Supplementary file 1 [file genes-14-00821-s001.zip › Supplementary Figures.pdf]

Supplementary Figures

Model 1

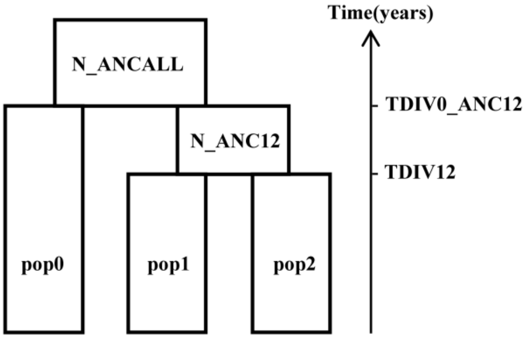

Model 2

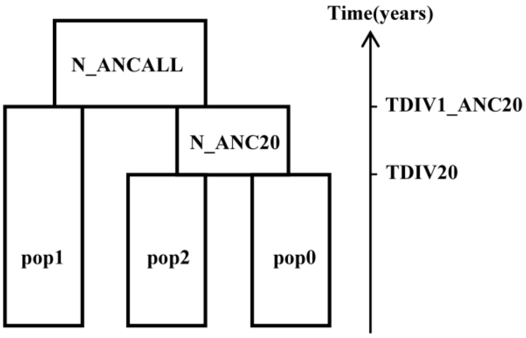

Model 3

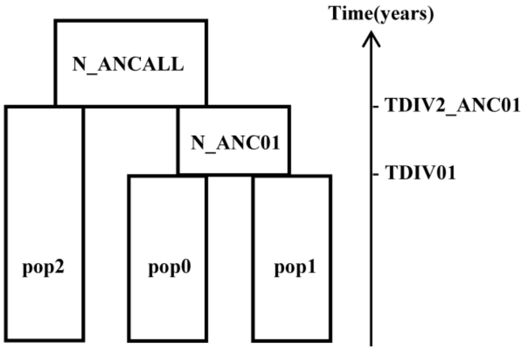

Model 4

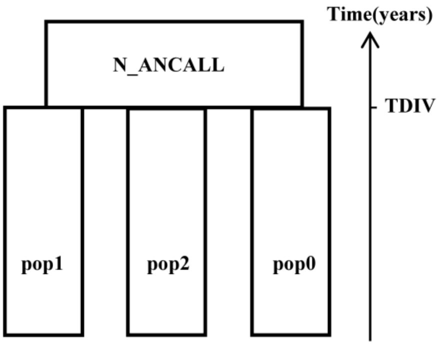

Model 5

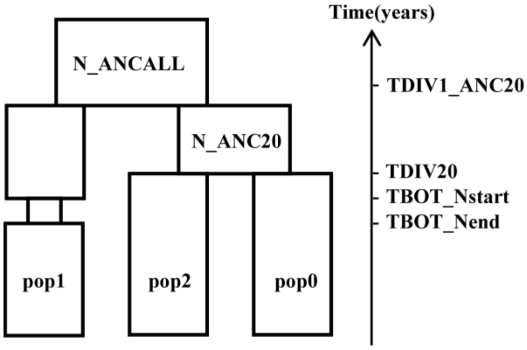

Model 6

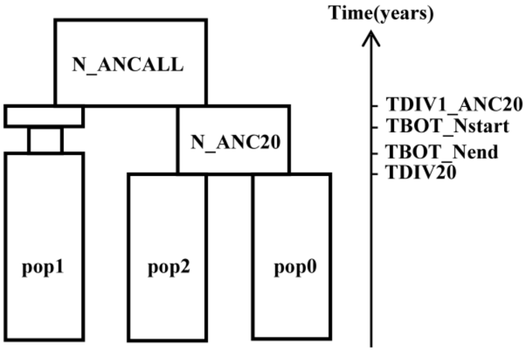

Model 7

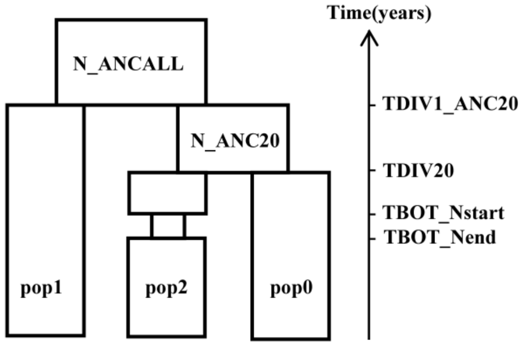

Model 8

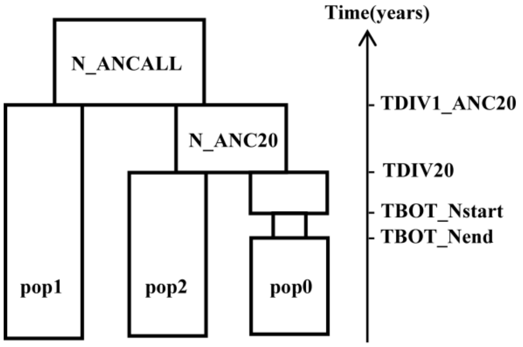

Model 9

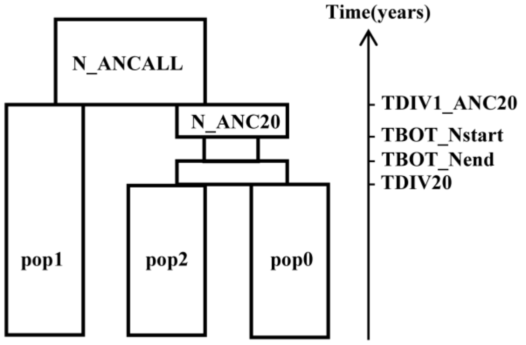

Model 10

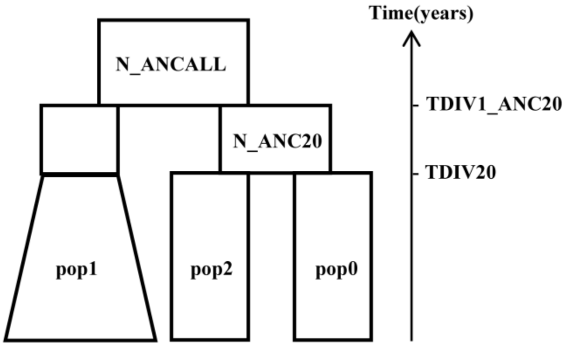

Model 11

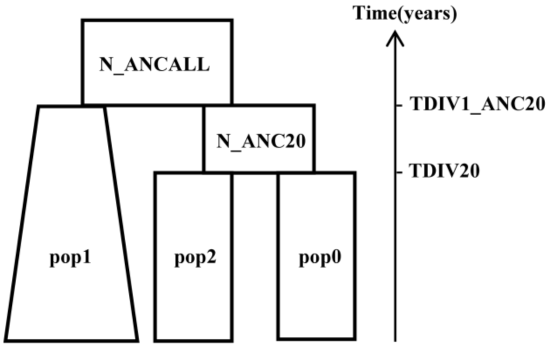

Model 12

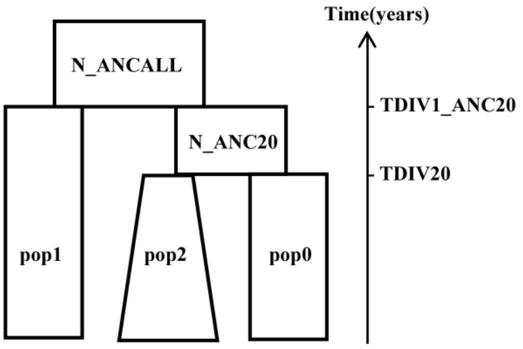

Model 13

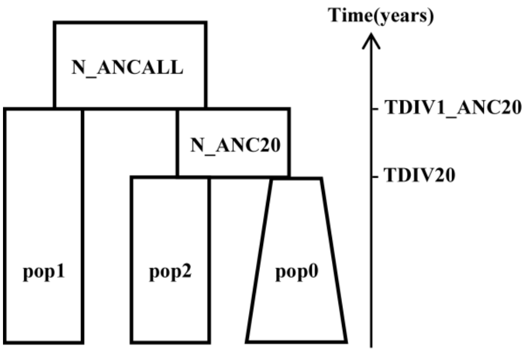

Model 14

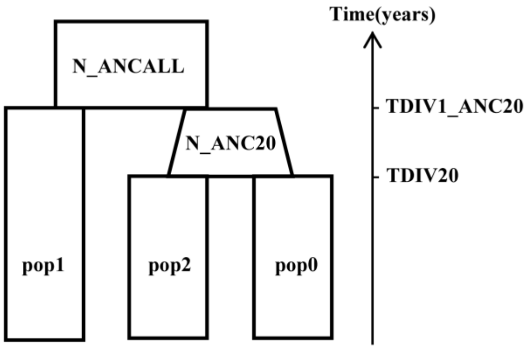

Model 15

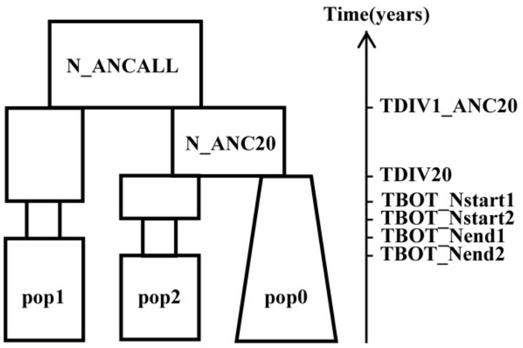

Model 16

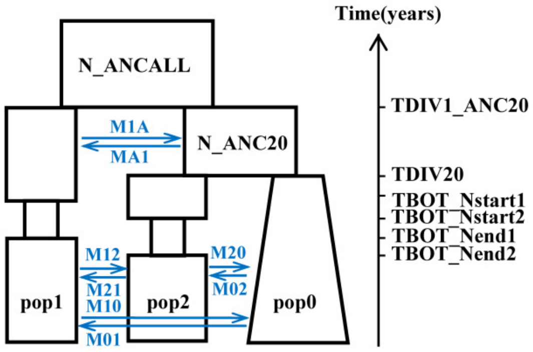

Model 17

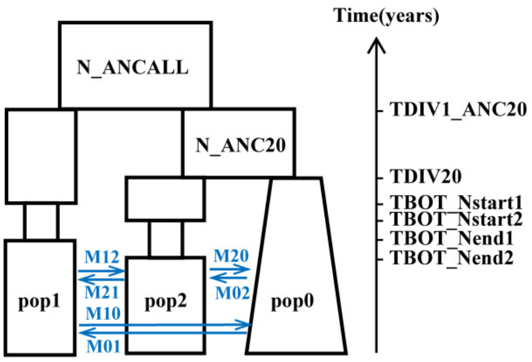

Model 18

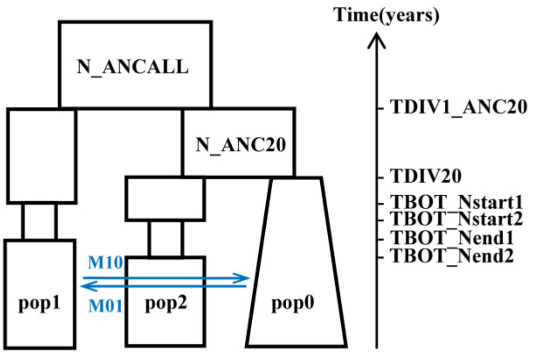

Model 19

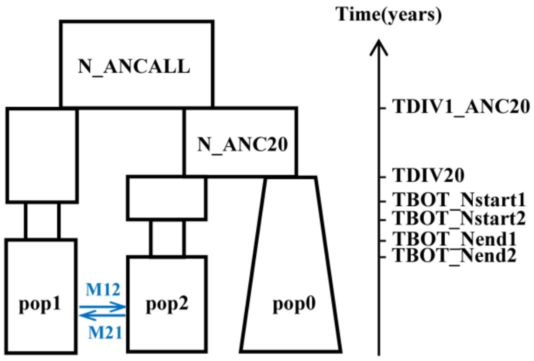

Model 20

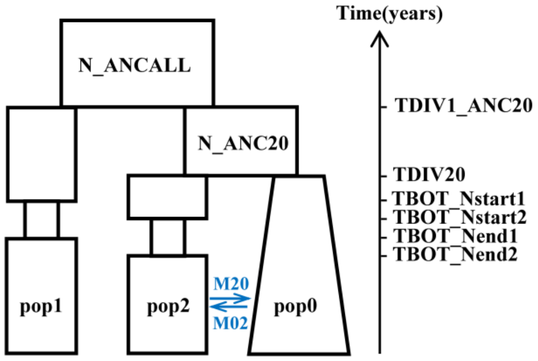

Model 21

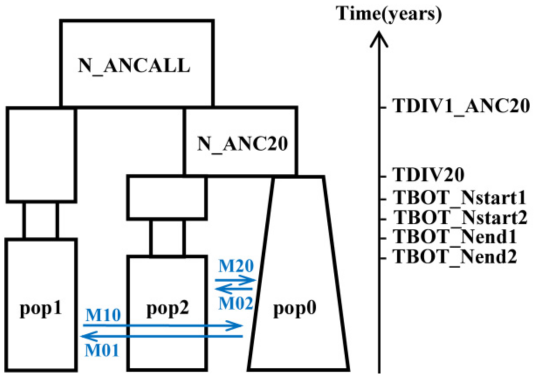

Figure S1. Tested demographic models. “pop0” represents the central population, “pop1” represents the southwest population, “pop2” represents the north population. Model 1, a two-step isolation of three populations without gene flow, N_ANCALL divided into pop0 and N_ANC12 in TDIV0_ANC12, N_ANC12 divided into pop1 and pop2 in TDIV12; Model 2, a two-step isolation of three populations without gene flow, N_ANCALL divided into pop1 and N_ANC20 in TDIV1_ANC20, N_ANC20 divided into pop2 and pop0 in TDIV20; Model 3, a two-step isolation of three populations without gene flow, N_ANCALL divided into pop2 and N_ANC01 in TDIV2_ANC01, N_ANC01 divided into pop0 and pop1 in TDIV01; Model 4, isolation of three populations without gene flow, N_ANCALL divided into pop0, pop1 and pop2 in TDIV. Based on Model 2, further tested demographic models. Model 5 ~ Model 9, a two-step isolation of three populations without gene flow, in which one population experienced three steps of stepwise population size changes, the difference between models is the different population and the time compared to TDIV20; Model 10 ~ Model 14, a two-step isolation of three populations without gene flow, in which one population experienced exponential population size changes, the difference between models is the different population and the time compared to TDIV20; Model 15, a two-step isolation of three populations without gene flow, in which pop1 and pop2 experienced three steps of stepwise population size changes,

pop0 experienced exponential population size changes, after TDIV20; based on Model 15, further tested demographic models. Model 16 ~ Model 21, a two-step isolation of three populations with asymmetric gene flow, the difference between models is the occurrence and the time of gene flow between different populations; Model 22, a two-step isolation of three populations, in which pop1 and pop2 experienced three steps of stepwise population size changes, with asymmetric gene flow between the three populations after TDIV20.

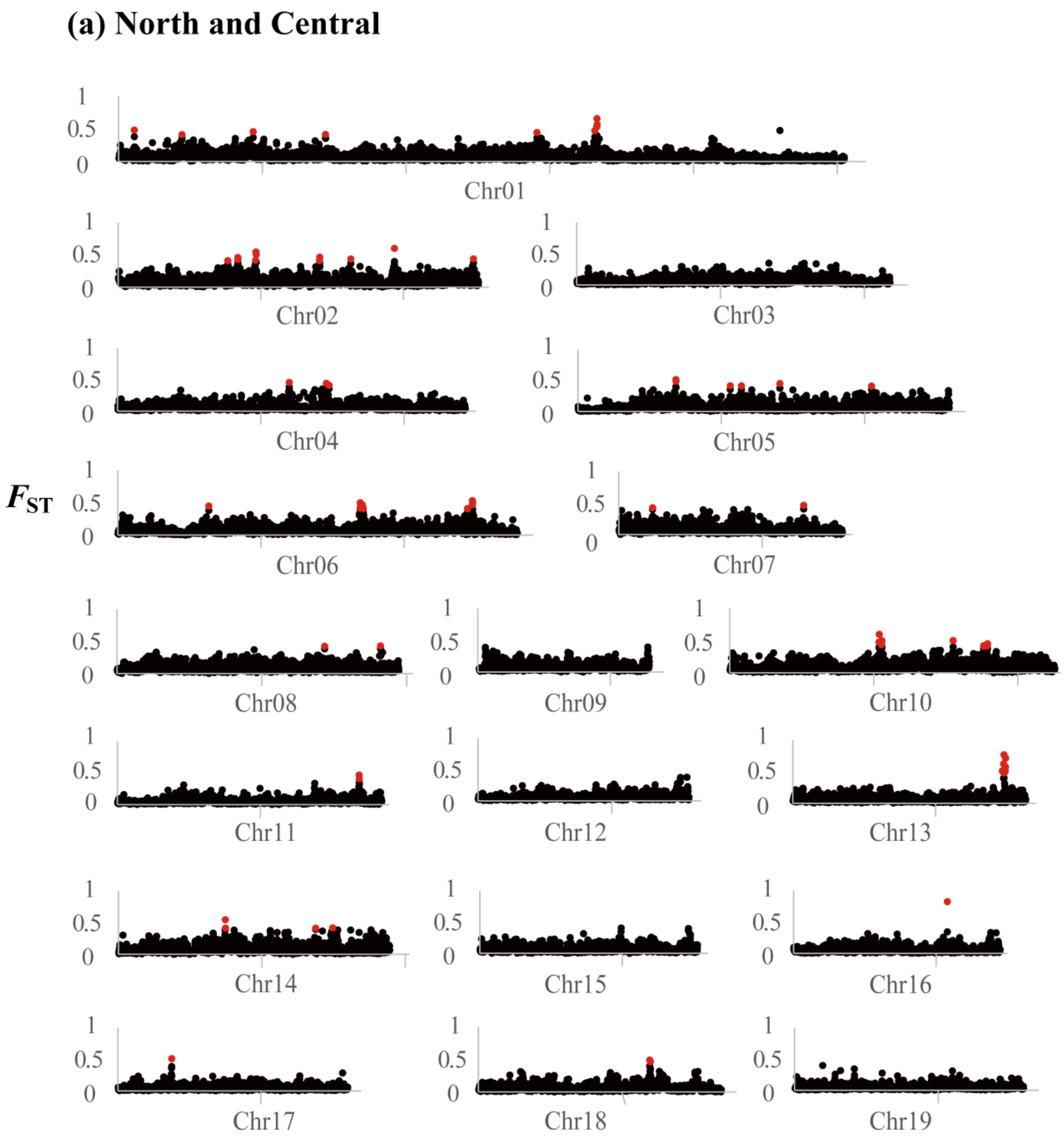

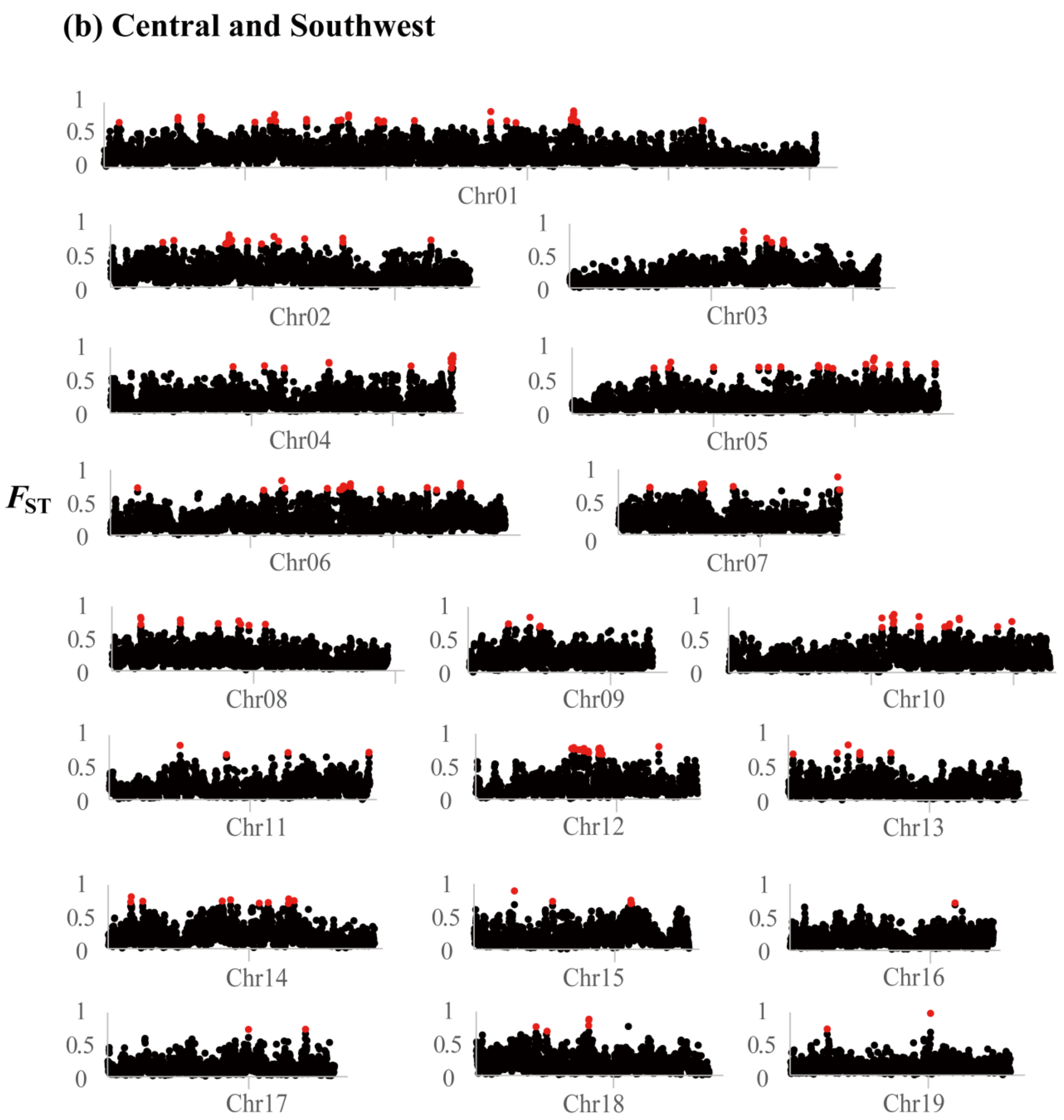

Figure S2. Genome-wide divergence of the chromosome. (a) The chromosomal distribution of genetic divergence (F_{ST}) between the north and central populations in 10-kbp non-overlapping windows; (b) the chromosomal distribution of genetic divergence (F_{ST}) between the central and southwest populations in 10-kbp non-overlapping windows. Windows with significantly high genetic differentiation are shown in red, and background windows are shown in black.

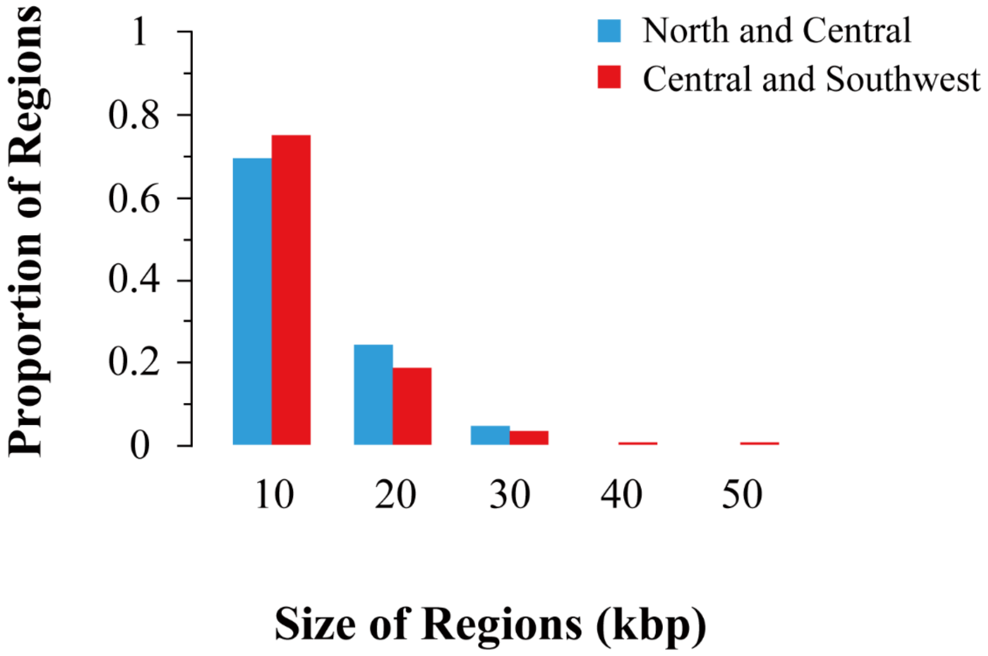

Figure S3. The physical size distributions (kbp) of regions displaying significantly high genetic differentiation. The north and central populations of *Populus davidiana* are shown in blue bars, and the central and southwest populations are shown in red bars.

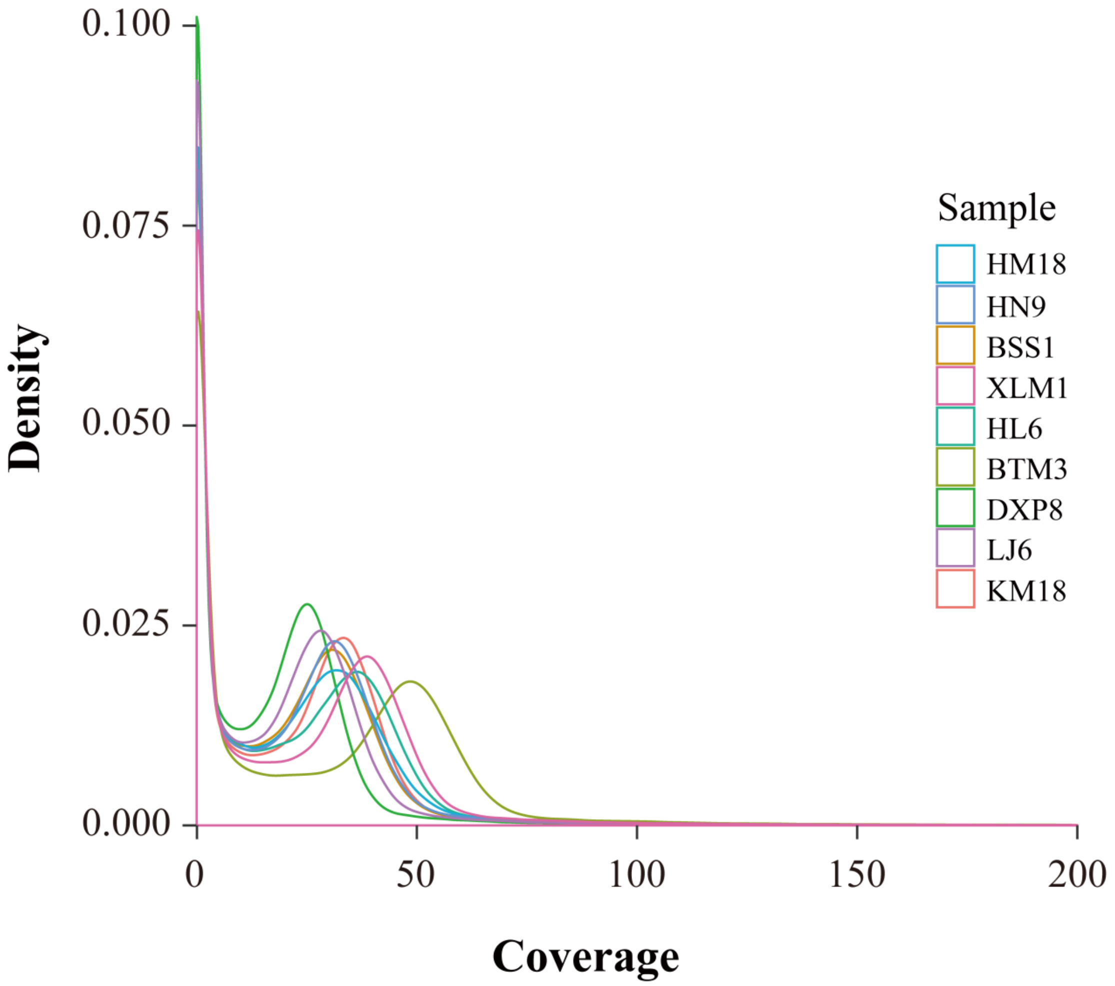

Figure S4. The distributions of the reads. HM18, HN9 and BSS1 are from the north population; XLM1, HL6 and BTM3 are from the central population; DXP8, LJ6 and KM18 are from the southwest population.

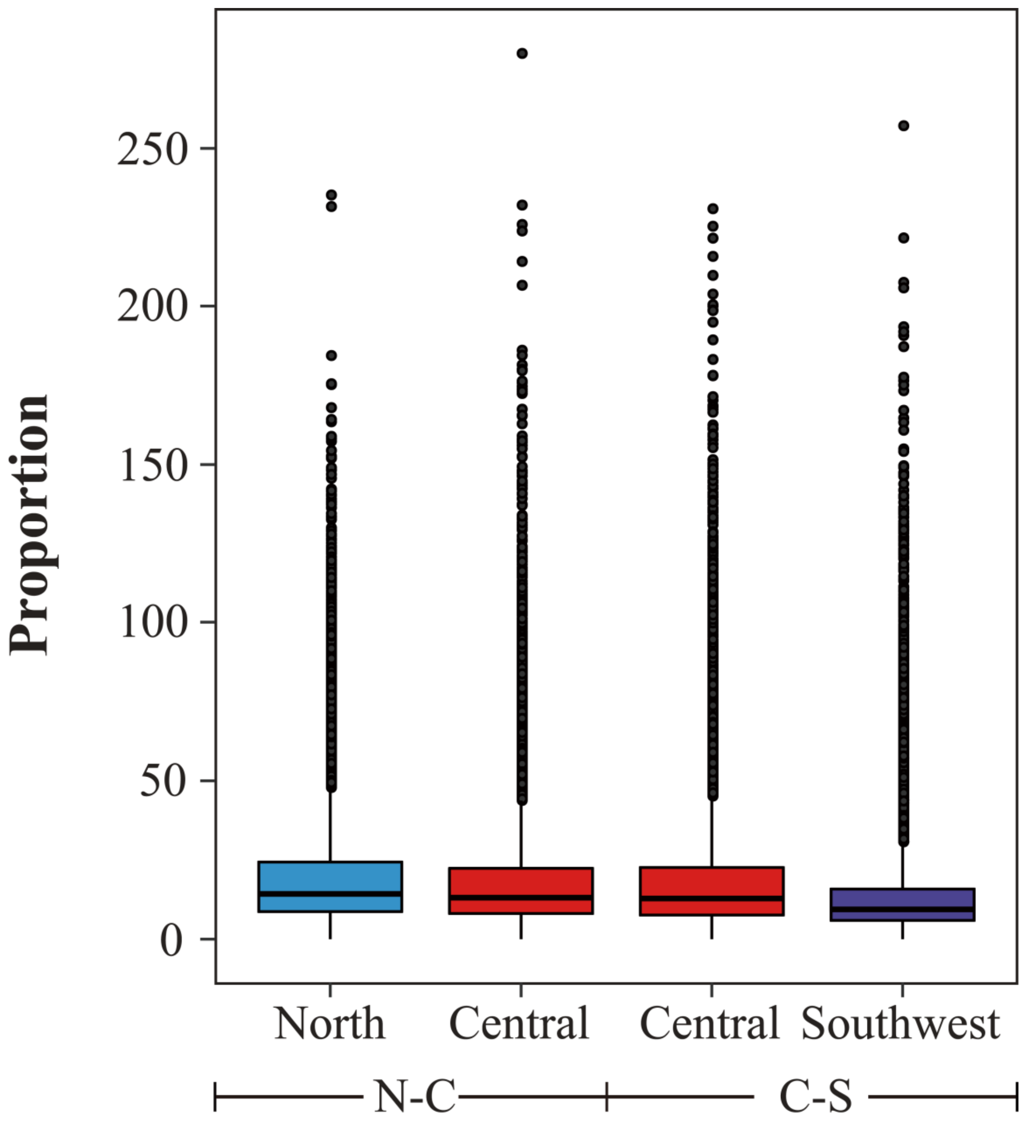

Figure S5. The proportions of ancestral state bases/derived bases across the whole genome. N-C represents parameters in the north and central populations, C-S represents parameters in the central and southwest populations.

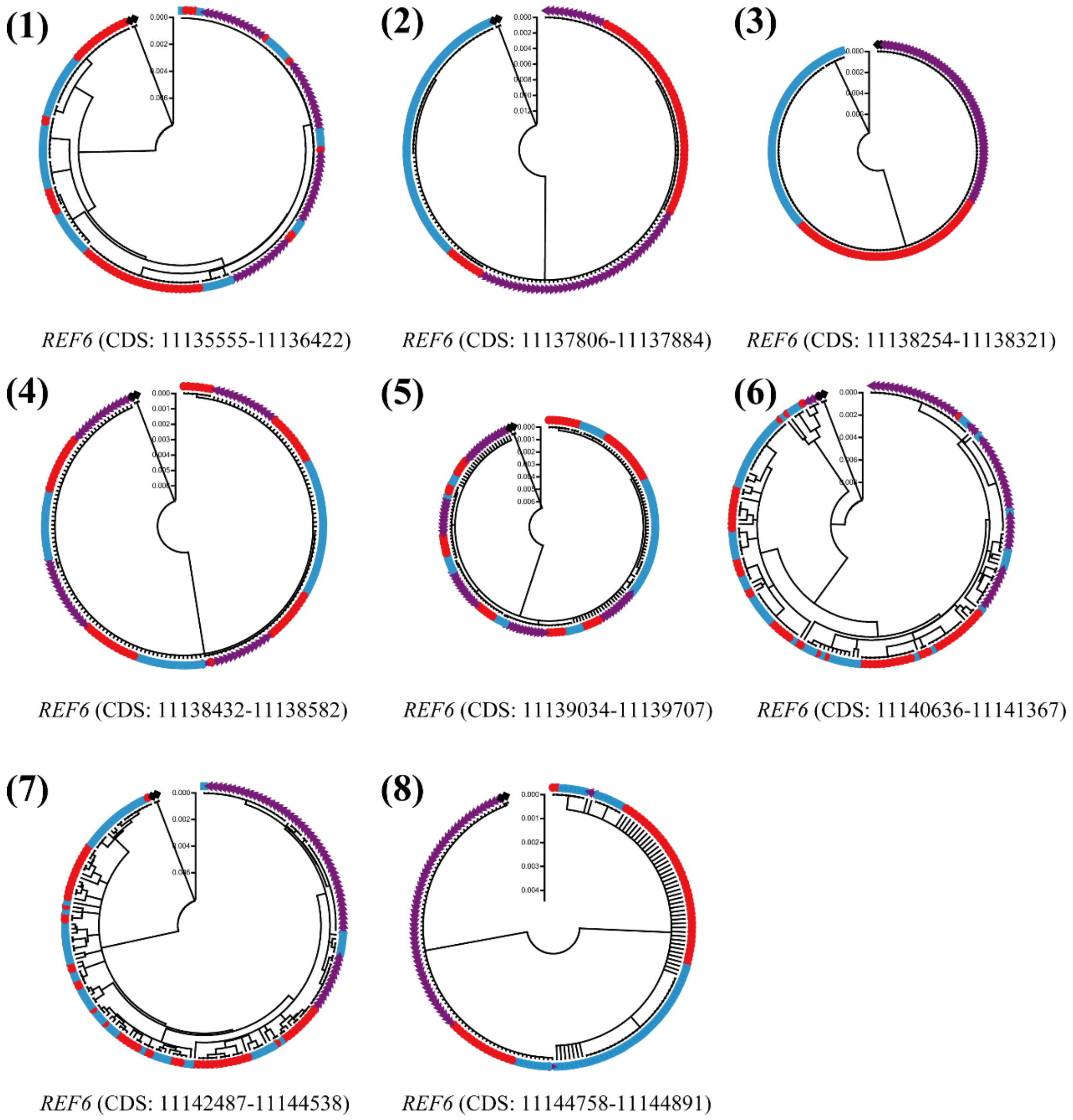

Figure S6. Phylogenetic tree of the combined DNA haplotypes. A1 ~ A8 are the eight CDS intervals of the *REF6* gene. Rhombus (black) represents the outgroup (*Populus trichocarpa*), square (blue) represents the north population, rotundity (red) represents the central population, triangle (purple) represents the southwest population.
