# Supplementary material for: A Genomic Quantitative Study on the Contribution of the Ancestral-State Bases Relative to Derived Bases in the Divergence and Local Adaptation of Populus davidiana
Source: Genes (Basel). 2023 Mar 29;14(4):821. doi: 10.3390/genes14040821 (PMC10137690; doi:10.3390/genes14040821)
Supplement: Supplementary file 1 [file genes-14-00821-s001.zip › Supplementary Table S1-S7(except Table S2b,S2c).pdf]

Supplementary Tables

Table S1. Overview information of Illumina re-sequencing data per sample of *Populus davidiana*.

Sample ID	Location	Latitude	Longitude	Mapping rate (%)	Mean Coverage
**North**					
HM8	Heilongjiang	51.7398	126.6566	85.38%	25.5492
HM18	Heilongjiang	51.7343	126.6750	87.01%	26.7308
HM21	Heilongjiang	51.7293	126.6612	83.61%	27.6120
HM23	Heilongjiang	51.7231	126.6566	84.57%	34.5065
HH1	Heilongjiang	50.2557	127.5166	85.14%	28.0336
HH2	Heilongjiang	50.2503	127.5414	84.23%	26.5943
HH12	Heilongjiang	50.2410	127.5435	84.88%	25.3972
HH16	Heilongjiang	50.2351	127.5224	85.19%	31.0539
HH23	Heilongjiang	50.2330	127.5076	86.32%	23.0257
SYS30	Heilongjiang	46.6521	131.1886	84.61%	20.6160
SYS33	Heilongjiang	46.6432	131.1714	85.32%	23.5610
HN2	Heilongjiang	46.2669	130.5964	84.82%	24.7351
HN9	Heilongjiang	46.2276	130.5346	84.31%	26.1190
LHS3	Jilin	43.8972	125.5465	86.03%	25.7924
LHS8	Jilin	43.8967	125.5591	85.70%	26.7667
LHS14	Jilin	43.8716	125.8380	85.50%	38.1427
LHS15	Jilin	43.8817	125.5506	85.18%	22.8187
SS1	Jilin	43.7027	123.9385	83.65%	21.5635
SS15	Jilin	43.6943	123.9190	85.92%	33.8928
SS18	Jilin	43.6926	123.8781	84.87%	20.9121
SS24	Jilin	43.6907	123.8518	86.14%	18.5633
BSS1	Jilin	43.5914	127.5697	85.14%	25.9662
BSS2	Jilin	43.5833	127.5693	84.39%	23.1904
BSS15	Jilin	43.5764	127.5736	82.99%	35.3335
BSS18	Jilin	43.5662	127.5679	84.77%	37.2733
BSS20	Jilin	43.5545	127.5666	83.65%	30.8521
JSZ17	Jilin	43.1700	126.8245	85.47%	22.3994
JSZ18	Jilin	43.1621	126.8175	83.98%	23.6758
JSZ22	Jilin	43.1505	126.8074	83.90%	26.8231
JSZ25	Jilin	43.1422	126.7857	84.94%	36.0175
Mean				84.92%	27.1173
**Central**					
WC1	Hebei	41.9506	117.7609	84.17%	30.0849
WC2	Hebei	41.9368	117.7639	84.16%	27.2286
WC8	Hebei	41.9281	117.7670	82.57%	24.4892

XLM1	Beijing	39.9691	115.4722	84.45%	31.2666
XLM2	Beijing	39.9735	115.4723	83.51%	33.3467
XLM21	Beijing	39.9750	115.4790	82.90%	29.6745
XLM22	Beijing	39.9689	115.4822	83.38%	29.1875
XLM23	Beijing	39.9660	115.4838	83.19%	30.0837
HL1	Beijing	39.9776	116.0702	84.25%	30.0600
HL2	Beijing	39.9761	116.0767	84.06%	32.5832
HL5	Beijing	39.9754	116.0812	82.94%	27.4777
HL6	Beijing	39.9725	116.0846	83.57%	28.5164
HL7	Beijing	39.9709	116.0932	83.99%	23.4592
HL8	Beijing	39.9699	116.1003	83.30%	23.9004
WT1	Shanxi	38.9858	113.5825	85.45%	27.2519
WT2	Shanxi	38.9809	113.5841	85.13%	29.4578
WT16	Shanxi	38.9776	113.5842	85.72%	25.8835
GDS1	Shanxi	37.8856	111.5516	85.83%	24.2600
GDS5	Shanxi	37.8818	111.5556	83.53%	31.3641
GDS8	Shanxi	37.8776	111.5590	85.30%	26.6884
GDS19	Shanxi	37.8727	111.5591	84.74%	27.6472
GDS27	Shanxi	37.8691	111.5563	85.81%	28.9126
GS2	Gansu	34.5972	105.7400	84.23%	31.6469
HeN1	Henan	34.3105	111.0871	84.19%	34.3747
HeN2	Henan	34.3074	111.0748	84.66%	28.6066
BTM3	Henan	33.4056	111.9983	85.20%	37.2249
BTM17	Henan	33.3795	111.9517	84.92%	30.7627
WS1	Chongqing	31.1029	109.8972	84.30%	25.4904
WS2	Chongqing	31.0734	109.8776	85.26%	22.5582
Mean				84.30%	28.7410
**Southwest**					
DXP2	Sichuan	27.5275	102.3197	84.70%	31.7036
DXP4	Sichuan	27.5323	102.3195	84.68%	30.2657
DXP5	Sichuan	27.5294	102.3201	81.96%	22.2481
DXP8	Sichuan	27.5259	102.3172	82.32%	21.3713
JBG1	Sichuan	27.4273	102.1368	81.39%	32.0898
JBG2	Sichuan	27.4244	102.1233	84.99%	27.6572
JBG3	Sichuan	27.4265	102.1182	84.03%	32.1805
WN1	Guizhou	26.9049	104.2931	84.46%	33.5209
WN4	Guizhou	26.8886	104.2765	84.19%	31.6999
LJ1	Yunnan	26.8897	100.3083	83.93%	22.0907
LJ2	Yunnan	26.8852	100.3004	84.17%	21.9906
LJ3	Yunnan	26.8792	100.3042	84.38%	36.9759
LJ4	Yunnan	26.8536	100.3045	85.28%	24.0629
LJ5	Yunnan	26.8454	100.3067	83.50%	23.8363
LJ6	Yunnan	26.8268	100.3093	84.37%	24.3539

LJ7	Yunnan	26.8159	100.3098	85.03%	25.9548
LJ8	Yunnan	26.7980	100.3092	83.81%	21.6704
LJ9	Yunnan	26.7861	100.3023	84.19%	24.1840
JC6	Yunnan	26.5505	99.9192	83.72%	24.5643
JC13	Yunnan	26.5449	99.9101	81.52%	26.9000
DL2	Yunnan	25.7103	100.3680	83.93%	26.6903
DL3	Yunnan	25.7053	100.3644	83.98%	30.2712
DL13	Yunnan	25.6992	100.3634	81.66%	22.7873
DL14	Yunnan	25.6918	100.3617	82.35%	28.2417
DL16	Yunnan	25.6805	100.3588	81.92%	26.6723
KM1	Yunnan	24.8305	102.8719	84.37%	31.9678
KM3	Yunnan	24.8284	102.8613	84.02%	30.5924
KM11	Yunnan	24.8224	102.8517	85.04%	22.8253
KM12	Yunnan	24.8191	102.8439	84.84%	20.9141
KM18	Yunnan	24.8146	102.8334	82.43%	26.0161
KM19	Yunnan	24.8101	102.8216	81.92%	27.3672
Mean				83.65%	26.8925

Table S2. Parameter settings and results for 21 demographic models associated with Figure S1.

(a) The setting ranges of the parameter estimation. “pop0” represents central population, “pop1” represents southwest population, “pop2” represents north population.

Model 1

[PARAMETERS]
//#isInt? #name #dist.#min #max
//all Ns are in number of haploid individuals
1 N_POP0 unif 1e4 5e6 output
1 N_POP1 unif 1e4 5e6 output
1 N_POP2 unif 1e4 5e6 output
1 N_ANCAll unif 1e4 5e6 output
1 N_ANC12 unif 1e4 5e6 output
1 TDIV12 unif 1e4 1e6 output
1 TPLUS12 unif 1e4 1e6 hide
[RULES]
[COMPLEX PARAMETERS]
1 TDIV0_ANC12 = TDIV12+TPLUS12 output
0 RELANC12 = N_ANC12/N_POP2 hide
0 RELANC02 = N_ANCAll/N_ANC12 hide

Model 2

[PARAMETERS]
//#isInt? #name #dist.#min #max
//all Ns are in number of haploid individuals
1 N_POP0 unif 1e4 5e6 output
1 N_POP1 unif 1e4 5e6 output
1 N_POP2 unif 1e4 5e6 output
1 N_ANCAll unif 1e4 5e6 output
1 N_ANC20 unif 1e4 5e6 output
1 TDIV20 unif 1e4 1e6 output
1 TPLUS20 unif 1e4 1e6 hide
[RULES]
[COMPLEX PARAMETERS]
1 TDIV1_ANC20 = TDIV20+TPLUS20 output
0 RELANC20 = N_ANC20/N_POP0 hide
0 RELANC10 = N_ANCAll/N_ANC20 hide

Model 3

[PARAMETERS]
//#isInt? #name #dist.#min #max
//all Ns are in number of haploid individuals
1 N_POP0 unif 1e4 5e6 output

```
1   N_POP1          unif    1e4    5e6   output
1   N_POP2          unif    1e4    5e6   output
1   N_ANCAll        unif    1e4    5e6   output
1   N_ANC01         unif    1e4    5e6   output
1   TDIV01          unif    1e4    1e6   output
1   TPLUS01         unif    1e4    1e6   hide
[RULES]
[COMPLEX PARAMETERS]
1   TDIV2_ANC01    = TDIV01+TPLUS01   output
0   RELANC01 = N_ANC01/N_POP1         hide
0   RELANC21 = N_ANCAll/N_ANC01       hide
```

Model 4

```
[PARAMETERS]
//#isInt? #name     #dist.#min   #max
//all Ns are in number of haploid individuals
1   N_POP0          unif    1e4    5e6   output
1   N_POP1          unif    1e4    5e6   output
1   N_POP2          unif    1e4    5e6   output
1   N_ANCAll        unif    1e4    5e6   output
1   TDIV            unif    1e4    1e6   output
[RULES]
[COMPLEX PARAMETERS]
0   RELANC = N_ANC/N_POP0      hide
```

Model 5

```
[PARAMETERS]
//#isInt? #name     #dist.#min   #max
//all Ns are in number of haploid individuals
1   N_POP0          unif    1e4    5e6   output
1   N_POP1          unif    1e4    5e6   output
1   N_POP2          unif    1e4    5e6   output
1   N_ANCAll        unif    1e4    5e6   output
1   N_ANC20         unif    1e4    5e6   output
0   RELstartBOT     unif    1e-4   1     hide    bounded
1   N_ancPOP1       unif    1      1e6   output
0   multTbot        unif    1e-4   1     hide    bounded
1   TDIV20          unif    1e4    1e6   output
1   TPLUS20         unif    1e4    1e6   hide
[RULES]
[COMPLEX PARAMETERS]
1   TDIV1_ANC20    = TDIV20+TPLUS20      output
1   TforBotN       = TDIV20-1000         hide
```

```
1    AuxforBotN    = TforBotN*multTbot            hide
1    TBOT_Nend      = 1000+AuxforBotN          output
1    TBOT_Nstart = TBOT_Nend-1000              output
1    N_BOT1         = RELstartBOT*N_POP1     output
0    RELendBOT    = N_ancPOP1/N_BOT1        hide
0    RELANC10 = N_ANCAll/N_ANC20           hide
0    RELANC20 = N_ANC20/N_POP0              hide
```

Model 6

```
[PARAMETERS]
//#isInt? #name      #dist.#min    #max
//all Ns are in number of haploid individuals
1    N_POP0          unif      1e4     5e6     output
1    N_POP1          unif      1e4     5e6     output
1    N_POP2          unif      1e4     5e6     output
1    N_ANCAll        unif      1e4     5e6     output
1    N_ANC20         unif      1e4     5e6     output
0    RELstartBOT     unif      1e-4    1       hide     bounded
1    N_ancPOP1       unif      1       1e6     output
0    multTbot        unif      1e-4    1       hide     bounded
1    TDIV20          unif      1e4     1e6     output
1    TPLUS20         unif      1e4     1e6     hide
[RULES]
[COMPLEX PARAMETERS]
1    TDIV1_ANC20    = TDIV20+TPLUS20    output
1    TPLUS21 = TPLUS20-1000                  hide
1    AuxforBotN = TPLUS21*multTbot           hide
1    TBOT_Nstart = TDIV20+AuxforBotN        output
1    TBOT_Nend = TBOT_Nstart+1000           output
1    N_BOT1        = RELstartBOT*N_POP1      output
0    RELendBOT = N_ancPOP1/N_BOT1          hide
0    RELANC10 = N_ANCAll/N_ANC20           hide
0    RELANC20 = N_ANC20/N_POP0           hide
```

Model 7

```
[PARAMETERS]
//#isInt? #name      #dist.#min    #max
//all Ns are in number of haploid individuals
1    N_POP0          unif      1e4     5e6     output
1    N_POP1          unif      1e4     5e6     output
1    N_POP2          unif      1e4     5e6     output
1    N_ANCAll        unif      1e4     5e6     output
1    N_ANC20         unif      1e4     5e6     output
```

```
0   RELstartBOT   unif   1e-4   1     hide     bounded
1   N_ancPOP2     unif   1      1e6   output
0   multTbot      unif   1e-4   1     hide     bounded
1   TDIV20        unif   1e4    1e6   output
1   TPLUS20       unif   1e4    1e6   hide
[RULES]
[COMPLEX PARAMETERS]
1   TDIV1_ANC20   = TDIV20+TPLUS20          output
1   TforBotN      = TDIV20-1000             hide
1   AuxforBotN    = TforBotN*multTbot       hide
1   TBOT_Nend     = 1000+AuxforBotN         output
1   TBOT_Nstart = TBOT_Nend-1000            output
1   N_BOT2        = RELstartBOT*N_POP2      output
0   RELendBOT     = N_ancPOP2/N_BOT2        hide
0   RELANC10 = N_ANCAll/N_ANC20             hide
0   RELANC20 = N_ANC20/N_POP0               hide
```

Model 8

```
[PARAMETERS]
//#isInt? #name   #dist.#min   #max
//all Ns are in number of haploid individuals
1   N_POP0        unif   1e4    5e6   output
1   N_POP1        unif   1e4    5e6   output
1   N_POP2        unif   1e4    5e6   output
1   N_ANCAll      unif   1e4    5e6   output
1   N_ANC20       unif   1e4    5e6   output
0   RELstartBOT   unif   1e-4   1     hide     bounded
1   N_ancPOP0     unif   1      1e6   output
0   multTbot      unif   1e-4   1     hide     bounded
1   TDIV20        unif   1e4    1e6   output
1   TPLUS20       unif   1e4    1e6   hide
[RULES]
[COMPLEX PARAMETERS]
1   TDIV1_ANC20   = TDIV20+TPLUS20          output
1   TforBotN      = TDIV20-1000             hide
1   AuxforBotN    = TforBotN*multTbot       hide
1   TBOT_Nend     = 1000+AuxforBotN         output
1   TBOT_Nstart = TBOT_Nend-1000            output
1   N_BOT0        = RELstartBOT*N_POP0      output
0   RELendBOT     = N_ancPOP0/N_BOT0        hide
0   RELANC10 = N_ANCAll/N_ANC20             hide
0   RELANC20 = N_ANC20/N_POP0               hide
```

Model 9

```
[PARAMETERS]
//#isInt? #name      #dist.#min    #max
//all Ns are in number of haploid individuals
1    N_POP0          unif        1e4       5e6     output
1    N_POP1          unif        1e4       5e6     output
1    N_POP2          unif        1e4       5e6     output
1    N_ANCAll        unif        1e4       5e6     output
1    N_ANC20         unif        1e4       5e6     output
0    RELstartBOT     unif        1e-4      1       hide      bounded
1    N_ancPOP0       unif        1         1e6     output
0    multTbot        unif        1e-4      1       hide      bounded
1    TDIV20          unif        1e4       1e6     output
1    TPLUS20         unif        1e4       1e6     hide
[RULES]
[COMPLEX PARAMETERS]
1    TDIV1_ANC20    = TDIV20+TPLUS20      output
1    TPLUS21 = TPLUS20-1000               hide
1    AuxforBotN = TPLUS21*multTbot        hide
1    TBOT_Nstart = TDIV20+AuxforBotN      output
1    TBOT_Nend = TBOT_Nstart+1000         output
1    N_BOT0       = RELstartBOT*N_POP0    output
0    RELendBOT = N_ancPOP0/N_BOT0         hide
0    RELANC10 = N_ANCAll/N_ANC20          hide
0    RELANC20 = N_ANC20/N_POP0            hide
```

Model 10

```
[PARAMETERS]
//#isInt? #name      #dist.#min    #max
//all Ns are in number of haploid individuals
1    N_POP0          unif        1e4       5e6     output
1    N_POP1          unif        1e4       5e6     output
1    N_POP2          unif        1e4       5e6     output
1    N_ANCAll        unif        1e4       5e6     output
1    N_ANC20         unif        1e4       5e6     output
0    N1RESIZE        unif        1e-4      1       hide
1    TDIV20          unif        1e4       1e6     output
1    TPLUS20         unif        1e4       1e6     hide
[RULES]
[COMPLEX PARAMETERS]
1    TDIV1_ANC20    = TDIV20+TPLUS20      output
1    N1atSPLIT     =    N_POP1*N1RESIZE   output
0    RELANC10 = N_ANCAll/N_ANC20          hide
```

0 RELANC20 = N_ANC20/N_POP0 hide
0 tmpRATIOP1 = N1atSPLIT/N_POP1 hide
0 tmplogP1 = log(tmpRATIOP1) hide
0 GrowthP1 = tmplogP1/TDIV20 output

Model 11

[PARAMETERS]
//#isInt? #name #dist.#min #max
//all Ns are in number of haploid individuals
1 N_POP0 unif 1e4 5e6 output
1 N_POP1 unif 1e4 5e6 output
1 N_POP2 unif 1e4 5e6 output
1 N_ANCAll unif 1e4 5e6 output
1 N_ANC20 unif 1e4 5e6 output
0 N1RESIZE unif 1e-4 1 hide
1 TDIV20 unif 1e4 1e6 output
1 TPLUS20 unif 1e4 1e6 hide
[RULES]
[COMPLEX PARAMETERS]
1 TDIV1_ANC20 = TDIV20+TPLUS20 output
1 N1atSPLIT = N_POP1*N1RESIZE output
0 RELANC10 = N_ANCAll/N_ANC20 hide
0 RELANC20 = N_ANC20/N_POP0 hide
0 tmpRATIOP1 = N1atSPLIT/N_POP1 hide
0 tmplogP1 = log(tmpRATIOP1) hide
0 GrowthP1 = tmplogP1/TDIV20 output

Model 12

[PARAMETERS]
//#isInt? #name #dist.#min #max
//all Ns are in number of haploid individuals
1 N_POP0 unif 1e4 5e6 output
1 N_POP1 unif 1e4 5e6 output
1 N_POP2 unif 1e4 5e6 output
1 N_ANCAll unif 1e4 5e6 output
1 N_ANC20 unif 1e4 5e6 output
0 N2RESIZE unif 1e-4 1 hide
1 TDIV20 unif 1e4 1e6 output
1 TPLUS20 unif 1e4 1e6 hide
[RULES]
[COMPLEX PARAMETERS]
1 TDIV1_ANC20 = TDIV20+TPLUS20 output
1 N2atSPLIT = N_POP2*N2RESIZE output

```
0   RELANC10 = N_ANCAll/N_ANC20            hide
0   RELANC20 = N_ANC20/N_POP0              hide
0   tmpRATIOP2 = N2atSPLIT/N_POP2          hide
0   tmplogP2      = log(tmpRATIOP2)        hide
0   GrowthP2      = tmplogP2/TDIV20        output
```

Model 13

```
[PARAMETERS]
//#isInt? #name      #dist.#min    #max
//all Ns are in number of haploid individuals
1   N_POP0       unif     1e4     5e6    output
1   N_POP1       unif     1e4     5e6    output
1   N_POP2       unif     1e4     5e6    output
1   N_ANCAll     unif     1e4     5e6    output
1   N_ANC20      unif     1e4     5e6    output
0   N0RESIZE     unif     1e-4    1      hide
1   TDIV20       unif     1e4     1e6    output
1   TPLUS20      unif     1e4     1e6    hide
[RULES]
[COMPLEX PARAMETERS]
1   TDIV1_ANC20    = TDIV20+TPLUS20       output
1   N0atSPLIT      =    N_POP0*N0RESIZE   output
0   RELANC10 = N_ANCAll/N_ANC20           hide
0   RELANC20 = N_ANC20/N_POP0             hide
0   tmpRATIOP0 = N0atSPLIT/N_POP0         hide
0   tmplogP0      = log(tmpRATIOP0)       hide
0   GrowthP0      = tmplogP0/TDIV20       output
```

Model 14

```
[PARAMETERS]
//#isInt? #name      #dist.#min    #max
//all Ns are in number of haploid individuals
1   N_POP0       unif     1e4     5e6    output
1   N_POP1       unif     1e4     5e6    output
1   N_POP2       unif     1e4     5e6    output
1   N_ANCAll     unif     1e4     5e6    output
1   N_ANC20      unif     1e4     5e6    output
0   N0RESIZE     unif     1e-4    1      hide
1   TDIV20       unif     1e4     1e6    output
1   TPLUS20      unif     1e4     1e6    hide
[RULES]
[COMPLEX PARAMETERS]
1   TDIV1_ANC20    = TDIV20+TPLUS20     output
```

```
1   N0atSPLIT     =   N_POP0*N0RESIZE     output
0   RELANC10 = N_ANCAll/N_ANC20          hide
0   RELANC20 = N_ANC20/N_POP0            hide
0   tmpRATIOP0 = N0atSPLIT/N_POP0        hide
0   tmplogP0     = log(tmpRATIOP0)       hide
0   GrowthP0     = tmplogP0/TDIV20       output
```

Model 15

```
[PARAMETERS]
//#isInt? #name     #dist.#min    #max
//all Ns are in number of haploid individuals
1   N_ANCAll      unif     10     2e6    output
1   N_ANC20       unif     10     2e6    output
1   N_POP0        unif     10     2e6    output
1   N_POP1        unif     10     2e6    output
1   N_POP2        unif     10     2e6    output
1   N_BOT1        unif     1      1e6    output
1   N_BOT2        unif     1      1e6    output
1   N_ancPOP0     unif     10     2e6    output
0   N0RESIZE      unif     1e-4   1      hide
1   N_ancPOP1     unif     10     2e6    output
0   multTbot1     unif     1e-3   1      hide    bounded
1   N_ancPOP2     unif     10     2e6    output
0   multTbot2     unif     1e-3   1      hide    bounded
1   TDIV20        unif     10     8e4    output
1   TPLUS20       unif     10     8e4    hide
[RULES]
[COMPLEX PARAMETERS]
1   TDIV1_ANC20    = TDIV20+TPLUS20         output
1   TforBotN       = TDIV20-1000            hide
1   AuxforBotN1    = TforBotN*multTbot1     hide
1   TBOT_Nend1     = 1000+AuxforBotN1       output
1   TBOT_Nstart1 = TBOT_Nend1-1000          output
1   AuxforBotN2    = TforBotN*multTbot2     hide
1   TBOT_Nend2     = 1000+AuxforBotN2       output
1   TBOT_Nstart2 = TBOT_Nend2-1000          output
1   N0atSPLIT      = N_POP0*N0RESIZE        output
0   RELstartBOT1   = N_BOT1/N_POP1          hide
0   RELendBOT1     = N_ancPOP1/N_BOT1       hide
0   RELstartBOT2   = N_BOT2/N_POP2          hide
0   RELendBOT2     = N_ancPOP2/N_BOT2       hide
0   RELANC10 = N_ANCAll/N_ANC20             hide
0   RELANC20 = N_ANC20/N_ancPOP0            hide
```

```
0   tmpRATIOP0 = N0atSPLIT/N_POP0              hide
0   tmplogP0      = log(tmpRATIOP0)              hide
0   GrowthP0      = tmplogP0/TDIV20              output
```

Model 16

```
[PARAMETERS]
//#isInt? #name      #dist.#min    #max
//all Ns are in number of haploid individuals
1   N_POP0              unif        1e4       5e6     output
1   N_POP1              unif        1e4       5e6     output
1   N_POP2              unif        1e4       5e6     output
1   N_ANCAll            unif        1e4       5e6     output
1   N_ANC20             unif        1e4       5e6     output
1   N_ancPOP1           unif        1         1e6     output
1   N_ancPOP2           unif        1         1e6     output
0   multTbot1           unif        1e-4      1       hide     bounded
0   multTbot2           unif        1e-4      1       hide     bounded
0   RELstartBOT1        unif        1e-4      1       hide     bounded
0   RELstartBOT2        unif        1e-4      1       hide     bounded
0   N0RESIZE            unif        1e-4      1       hide
1   TDIV20              unif        1e4       1e6     output
1   TPLUS20             unif        1e4       1e6     hide
0   NM01                logunif     1e-5      5       hide bounded
0   NM10                logunif     1e-5      5       hide bounded
0   NM02                logunif     1e-5      5       hide bounded
0   NM20                logunif     1e-5      5       hide bounded
0   NM12                logunif     1e-5      5       hide bounded
0   NM21                logunif     1e-5      5       hide bounded
0   NM1A                logunif     1e-5      5       hide bounded
0   NMA1                logunif     1e-5      5       hide bounded
[RULES]
[COMPLEX PARAMETERS]
0   MIG01 =    NM01/N_POP0     output
0   MIG10 =    NM10/N_POP1     output
0   MIG02 =    NM02/N_POP0     output
0   MIG20 =    NM20/N_POP2     output
0   MIG12 =    NM12/N_POP1     output
0   MIG21 =    NM21/N_POP2     output
0   MIG1A =    NM1A/N_POP1     output
0   MIGA1 =    NMA1/N_ANC20 output
1   TDIV1_ANC20    = TDIV20+TPLUS20            output
1   TforBotN         = TDIV20-1000               hide
1   AuxforBotN1    = TforBotN*multTbot1         hide
```

```
1   TBOT_Nend1      = 1000+AuxforBotN1          output
1   TBOT_Nstart1 = TBOT_Nend1-1000              output
1   AuxforBotN2    = TforBotN*multTbot2          hide
1   TBOT_Nend2      = 1000+AuxforBotN2          output
1   TBOT_Nstart2 = TBOT_Nend2-1000              output
1   N0atSPLIT       = N_POP0*N0RESIZE            output
1   N_BOT1 = RELstartBOT1*N_POP1                output
0   RELendBOT1      = N_ancPOP1/N_BOT1          hide
1   N_BOT2 = RELstartBOT2*N_POP2                output
0   RELendBOT2      = N_ancPOP2/N_BOT2          hide
0   RELANC10 = N_ANCAll/N_ANC20                  hide
0   RELANC20 = N_ANC20/N_POP0                    hide
0   tmpRATIOP0 = N0atSPLIT/N_POP0               hide
0   tmplogP0      = log(tmpRATIOP0)              hide
0   GrowthP0      = tmplogP0/TDIV20              output
```

Model 17

```
[PARAMETERS]
//#isInt? #name      #dist.#min    #max
//all Ns are in number of haploid individuals
1   N_POP0          unif      1e4     5e6     output
1   N_POP1          unif      1e4     5e6     output
1   N_POP2          unif      1e4     5e6     output
1   N_ANCAll        unif      1e4     2e7     output
1   N_ANC20         unif      1e4     5e6     output
1   N_ancPOP1       unif      1       1e6     output
1   N_ancPOP2       unif      1       1e6     output
0   multTbot1       unif      1e-4    1       hide    bounded
0   multTbot2       unif      1e-4    1       hide    bounded
0   RELstartBOT1    unif      1e-4    1       hide    bounded
0   RELstartBOT2    unif      1e-4    1       hide    bounded
0   N0RESIZE        unif      1e-4    1       hide
1   TDIV20          unif      1e4     1e6     output
1   TPLUS20         unif      1e4     1e6     hide
0   NM01            logunif   1e-5    5       hide bounded
0   NM10            logunif   1e-5    5       hide bounded
0   NM02            logunif   1e-5    5       hide bounded
0   NM20            logunif   1e-5    5       hide bounded
0   NM12            logunif   1e-5    5       hide bounded
0   NM21            logunif   1e-5    5       hide bounded
[RULES]
[COMPLEX PARAMETERS]
0   MIG01 =    NM01/N_POP0    output
```

0 MIG10 = NM10/N_POP1 output
0 MIG02 = NM02/N_POP0 output
0 MIG20 = NM20/N_POP2 output
0 MIG12 = NM12/N_POP1 output
0 MIG21 = NM21/N_POP2 output
1 TDIV1_ANC20 = TDIV20+TPLUS20 output
1 TforBotN = TDIV20-1000 hide
1 AuxforBotN1 = TforBotN*multTbot1 hide
1 TBOT_Nend1 = 1000+AuxforBotN1 output
1 TBOT_Nstart1 = TBOT_Nend1-1000 output
1 AuxforBotN2 = TforBotN*multTbot2 hide
1 TBOT_Nend2 = 1000+AuxforBotN2 output
1 TBOT_Nstart2 = TBOT_Nend2-1000 output
1 N0atSPLIT = N_POP0*N0RESIZE output
1 N_BOT1 = RELstartBOT1*N_POP1 output
0 RELendBOT1 = N_ancPOP1/N_BOT1 hide
1 N_BOT2 = RELstartBOT2*N_POP2 output
0 RELendBOT2 = N_ancPOP2/N_BOT2 hide
0 RELANC10 = N_ANCAll/N_ANC20 hide
0 RELANC20 = N_ANC20/N_POP0 hide
0 tmpRATIOP0 = N0atSPLIT/N_POP0 hide
0 tmplogP0 = log(tmpRATIOP0) hide
0 GrowthP0 = tmplogP0/TDIV20 output

Model 18
[PARAMETERS]
//#isInt? #name #dist.#min #max
//all Ns are in number of haploid individuals
1 N_POP0 unif 1e4 5e6 output
1 N_POP1 unif 1e4 5e6 output
1 N_POP2 unif 1e4 5e6 output
1 N_ANCAll unif 1e4 5e6 output
1 N_ANC20 unif 1e4 5e6 output
1 N_ancPOP1 unif 1 1e6 output
1 N_ancPOP2 unif 1 1e6 output
0 multTbot1 unif 1e-4 1 hide bounded
0 multTbot2 unif 1e-4 1 hide bounded
0 RELstartBOT1 unif 1e-4 1 hide bounded
0 RELstartBOT2 unif 1e-4 1 hide bounded
0 N0RESIZE unif 1e-4 1 hide
1 TDIV20 unif 1e4 1e6 output
1 TPLUS20 unif 1e4 1e6 hide
0 NM01 logunif 1e-5 5 hide bounded

```
0   NM10              logunif      1e-5      5        hide bounded
[RULES]
[COMPLEX PARAMETERS]
0   MIG01 =    NM01/N_POP0    output
0   MIG10 =    NM10/N_POP1    output
1   TDIV1_ANC20    = TDIV20+TPLUS20          output
1   TforBotN         = TDIV20-1000                   hide
1   AuxforBotN1    = TforBotN*multTbot1            hide
1   TBOT_Nend1      = 1000+AuxforBotN1          output
1   TBOT_Nstart1 = TBOT_Nend1-1000              output
1   AuxforBotN2    = TforBotN*multTbot2            hide
1   TBOT_Nend2      = 1000+AuxforBotN2          output
1   TBOT_Nstart2 = TBOT_Nend2-1000              output
1   N0atSPLIT       = N_POP0*N0RESIZE          output
1   N_BOT1 = RELstartBOT1*N_POP1              output
0   RELendBOT1      = N_ancPOP1/N_BOT1       hide
1   N_BOT2 = RELstartBOT2*N_POP2              output
0   RELendBOT2      = N_ancPOP2/N_BOT2       hide
0   RELANC10 = N_ANCAll/N_ANC20              hide
0   RELANC20 = N_ANC20/N_POP0                 hide
0   tmpRATIOP0 = N0atSPLIT/N_POP0             hide
0   tmplogP0      = log(tmpRATIOP0)                hide
0   GrowthP0      = tmplogP0/TDIV20                output
```

Model 19

```
[PARAMETERS]
//#isInt? #name      #dist.#min    #max
//all Ns are in number of haploid individuals
1   N_POP0            unif        1e4       5e6     output
1   N_POP1            unif        1e4       5e6     output
1   N_POP2            unif        1e4       5e6     output
1   N_ANCAll          unif        1e4       5e6     output
1   N_ANC20           unif        1e4       5e6     output
1   N_ancPOP1        unif        1          1e6     output
1   N_ancPOP2        unif        1          1e6     output
0   multTbot1         unif        1e-4     1          hide     bounded
0   multTbot2         unif        1e-4     1          hide     bounded
0   RELstartBOT1    unif        1e-4     1          hide     bounded
0   RELstartBOT2    unif        1e-4     1          hide     bounded
0   N0RESIZE          unif        1e-4     1         hide
1   TDIV20             unif        1e4       1e6     output
1   TPLUS20           unif        1e4       1e6     hide
0   NM12              logunif       1e-5      5       hide bounded
```

0 NM21 logunif 1e-5 5 hide bounded
[RULES]
[COMPLEX PARAMETERS]
0 MIG12 = NM12/N_POP1 output
0 MIG21 = NM21/N_POP2 output
1 TDIV1_ANC20 = TDIV20+TPLUS20 output
1 TforBotN = TDIV20-1000 hide
1 AuxforBotN1 = TforBotN*multTbot1 hide
1 TBOT_Nend1 = 1000+AuxforBotN1 output
1 TBOT_Nstart1 = TBOT_Nend1-1000 output
1 AuxforBotN2 = TforBotN*multTbot2 hide
1 TBOT_Nend2 = 1000+AuxforBotN2 output
1 TBOT_Nstart2 = TBOT_Nend2-1000 output
1 N0atSPLIT = N_POP0*N0RESIZE output
1 N_BOT1 = RELstartBOT1*N_POP1 output
0 RELendBOT1 = N_ancPOP1/N_BOT1 hide
1 N_BOT2 = RELstartBOT2*N_POP2 output
0 RELendBOT2 = N_ancPOP2/N_BOT2 hide
0 RELANC10 = N_ANCAll/N_ANC20 hide
0 RELANC20 = N_ANC20/N_POP0 hide
0 tmpRATIOP0 = N0atSPLIT/N_POP0 hide
0 tmplogP0 = log(tmpRATIOP0) hide
0 GrowthP0 = tmplogP0/TDIV20 output

Model 20
[PARAMETERS]
//#isInt? #name #dist.#min #max
//all Ns are in number of haploid individuals
1 N_POP0 unif 1e4 5e6 output
1 N_POP1 unif 1e4 5e6 output
1 N_POP2 unif 1e4 5e6 output
1 N_ANCAll unif 1e4 5e6 output
1 N_ANC20 unif 1e4 5e6 output
1 N_ancPOP1 unif 1 1e6 output
1 N_ancPOP2 unif 1 1e6 output
0 multTbot1 unif 1e-4 1 hide bounded
0 multTbot2 unif 1e-4 1 hide bounded
0 RELstartBOT1 unif 1e-4 1 hide bounded
0 RELstartBOT2 unif 1e-4 1 hide bounded
0 N0RESIZE unif 1e-4 1 hide
1 TDIV20 unif 1e4 1e6 output
1 TPLUS20 unif 1e4 1e6 hide
0 NM02 logunif 1e-5 5 hide bounded

```
0   NM20                logunif         1e-5       5        hide bounded
[RULES]
[COMPLEX PARAMETERS]
0   MIG02 =    NM02/N_POP0    output
0   MIG20 =    NM20/N_POP2    output
1   TDIV1_ANC20    = TDIV20+TPLUS20           output
1   TforBotN         = TDIV20-1000                    hide
1   AuxforBotN1    = TforBotN*multTbot1            hide
1   TBOT_Nend1      = 1000+AuxforBotN1          output
1   TBOT_Nstart1 = TBOT_Nend1-1000              output
1   AuxforBotN2    = TforBotN*multTbot2            hide
1   TBOT_Nend2      = 1000+AuxforBotN2          output
1   TBOT_Nstart2 = TBOT_Nend2-1000            output
1   N0atSPLIT       = N_POP0*N0RESIZE          output
1   N_BOT1 = RELstartBOT1*N_POP1              output
0   RELendBOT1      = N_ancPOP1/N_BOT1       hide
1   N_BOT2 = RELstartBOT2*N_POP2              output
0   RELendBOT2      = N_ancPOP2/N_BOT2       hide
0   RELANC10 = N_ANCAll/N_ANC20              hide
0   RELANC20 = N_ANC20/N_POP0                hide
0   tmpRATIOP0 = N0atSPLIT/N_POP0            hide
0   tmplogP0      = log(tmpRATIOP0)                hide
0   GrowthP0      = tmplogP0/TDIV20                output
```

Model 21

```
[PARAMETERS]
//#isInt? #name      #dist.#min    #max
//all Ns are in number of haploid individuals
1   N_POP0              unif         1e4       5e6     output
1   N_POP1              unif         1e4       5e6     output
1   N_POP2              unif         1e4       5e6     output
1   N_ANCAll            unif         1e4       5e6     output
1   N_ANC20             unif         1e4       5e6     output
1   N_ancPOP1         unif         1           1e6     output
1   N_ancPOP2         unif         1           1e6     output
0   multTbot1          unif         1e-4       1         hide     bounded
0   multTbot2          unif         1e-4       1         hide     bounded
0   RELstartBOT1    unif         1e-4       1         hide     bounded
0   RELstartBOT2    unif         1e-4       1         hide     bounded
0   N0RESIZE           unif         1e-4       1         hide
1   TDIV20              unif         1e4       1e6     output
1   TPLUS20            unif         1e4       1e6     hide
0   NM01               logunif    1e-5       5        hide bounded
```

```
0    NM10              logunif    1e-5      5       hide bounded
0    NM02              logunif    1e-5      5       hide bounded
0    NM20              logunif    1e-5      5       hide bounded
[RULES]
[COMPLEX PARAMETERS]
0    MIG01 =    NM01/N_POP0    output
0    MIG10 =    NM10/N_POP1    output
0    MIG02 =    NM02/N_POP0    output
0    MIG20 =    NM20/N_POP2    output
1    TDIV1_ANC20    = TDIV20+TPLUS20            output
1    TforBotN         = TDIV20-1000                      hide
1    AuxforBotN1    = TforBotN*multTbot1          hide
1    TBOT_Nend1      = 1000+AuxforBotN1        output
1    TBOT_Nstart1 = TBOT_Nend1-1000            output
1    AuxforBotN2    = TforBotN*multTbot2          hide
1    TBOT_Nend2      = 1000+AuxforBotN2        output
1    TBOT_Nstart2 = TBOT_Nend2-1000            output
1    N0atSPLIT       = N_POP0*N0RESIZE          output
1    N_BOT1 = RELstartBOT1*N_POP1              output
0    RELendBOT1      = N_ancPOP1/N_BOT1      hide
1    N_BOT2 = RELstartBOT2*N_POP2              output
0    RELendBOT2      = N_ancPOP2/N_BOT2      hide
0    RELANC10 = N_ANCAll/N_ANC20              hide
0    RELANC20 = N_ANC20/N_POP0                hide
0    tmpRATIOP0 = N0atSPLIT/N_POP0            hide
0    tmplogP0      = log(tmpRATIOP0)                hide
0    GrowthP0      = tmplogP0/TDIV20                output
```

(d) Relative likelihood.

Model	Max(log10(Lhood$_i$)[a]	No. Of parameters(d)	AIC$_i$[b]	Δ_i[b]	Model normalized relative likelihood(w_i)[b]
Model 1	-183620336.094	7	845602911.321	48328356.603	~0
Model 2	-182950790.659	7	842519540.646	45244985.928	~0
Model 3	-183626375.203	7	845630722.446	48356167.728	~0
Model 4	-182958064.512	5	842553033.977	45278479.259	~0
Model 5	-182692586.123	10	841330470.815	44055916.097	~0
Model 6	-182786507.661	10	841762995.481	44488440.764	~0
Model 7	-182912941.285	10	842345243.837	45070689.119	~0
Model 8	-182950190.341	10	842516782.079	45242227.361	~0
Model 9	-183076857.076	10	843100103.951	45825549.233	~0
Model 10	-182800713.652	8	841828412.488	44553857.770	~0
Model 11	-182790253.257	8	841780240.588	44505685.871	~0
Model 12	-182949026.673	8	842511419.190	45236864.472	~0
Model 13	-182948113.875	8	842507215.600	45232660.882	~0
Model 14	-182961108.378	8	842567057.498	45292502.780	~0
Model 15	-182651175.982	14	841139778.068	43865223.350	~0
Model 16	-173153191.191	22	797399957.681	125402.964	~0
Model 17	-173125961.152	20	797274554.718	0	1
Model 18	-175100515.214	16	806367704.215	9093149.497	~0
Model 19	-175448902.218	16	807972085.659	10697530.941	~0
Model 20	-181643144.752	16	836497626.701	39223071.983	~0
Model 21	-173502427.952	18	799008244.401	1733689.683	~0

[a] Based on the best likelihood among the 50 independent runs for each model (Figure S1).

[b] The calculation of AIC$_i$, Δ_i and w_i are according to the methods shown in Excoffier *et al.* (2013).

Table S3. Summary population genetic statistics in the whole-genome of *Populus davidiana* (the mean±standard deviation values are displayed).

Parameters	Population	Whole-genome
ln ($\theta\pi \times 10^4$ +1)	North	4.3109(±0.8213)
	Central	4.2593(±0.8284)
	Southwest	4.0750(±0.8669)
Tajima's *D*	North	0.5385(±0.7286)
	Central	0.4208(±0.7764)
	Southwest	0.8467(±0.8996)
Fay & Wu's *H*	North	0.0109(±0.2479)
	Central	-0.0330(±0.2791)
	Southwest	-0.1821(±0.4332)
ln (r^2 +1)	North	0.1632(±0.0930)
	Central	0.1715(±0.1031)
	Southwest	0.2379(±0.1463)
recombination rate (ρ)	North	18.5441(±17.6823)
	Central	17.8010(±16.6714)
	Southwest	15.0683(±18.1228)
ln (Fixed +1) (%)	North	0.0043(±0.0067)
(derived bases)	Central (N-C)	0.0063(±0.0089)
	Central (C-S)	0.0067(±0.0110)
	Southwest	0.0175(±0.0225)
ln (Fixed +1) (%)	North	0.0530(±0.0247)
(ancestral-state bases)	Central (N-C)	0.0662(±0.0289)
	Central (C-S)	0.0661(±0.0294)
	Southwest	0.1150(±0.0471)
ln (Shared +1) (%)	North-Central	0.2462(±0.0185)
	Central-Southwest	0.2133(±0.0351)
F_{ST} (actual)	North-Central	0.0778(±0.0587)
	Central-Southwest	0.2066(±0.1372)
	North-Southwest	0.2331(±0.1377)
F_{ST} (simulated)	North-Central	0.0761(±0.0278)
	Central-Southwest	0.2018(±0.0593)
ln (d_{xy} +1)	North-Central	0.1731(±0.1568)
	Central-Southwest	0.2638(±0.2090)

Notes: Central (N-C) represent parameters of the central population of *P. davidiana* according to the North-Central populations, Central (C-S) represent parameters of the central population according to the Central-Southwest populations.

Table S4. Summary statistics comparing regions showing high genetic differentiation with other genomic regions of *Populus davidiana* (the mean±standard deviation values are displayed).

Parameters		Population	Regions displaying high differentiation	Background
ln (θπ ×10^4 +1)	N-C	North	3.4589(±1.2089)**	4.3126(±0.8195)
		Central	3.2355(±1.1049)***	4.2614(±0.8265)
	C-S	Central	3.4806(±0.8846)***	4.2634(±0.8261)
		Southwest	2.8751(±0.9217)***	4.0813(±0.8622)
Tajima's *D*	N-C	North	0.5706(±0.8835)	0.5224(±0.7193)
		Central	-0.0607(±1.0307)**	0.4015(±0.7660)
	C-S	Central	-0.2295(±0.9335)***	0.4039(±0.7645)
		Southwest	-0.6945(±0.9961)***	0.8483(±0.8935)
Fay & Wu's *H*	N-C	North	-0.4103(±0.4389)***	0.0118(±0.2467)
		Central	-0.5418(±0.4733)***	-0.0320(±0.2777)
	C-S	Central	-0.4514(±0.4156)***	-0.0308(±0.2766)
		Southwest	-1.1463(±0.7140)***	-0.1771(±0.4255)
ln (r^2 +1)	N-C	North	0.2635(±0.1418)**	0.1631(±0.0928)
		Central	0.3822(±0.1660)***	0.1712(±0.1026)
	C-S	Central	0.2540(±0.1418)***	0.1711(±0.1027)
		Southwest	0.2687(±0.1921)	0.2377(±0.1460)
ln (Fixed +1) (%) (derived bases)	N-C	North	0.0075(±0.0162)	0.0043(±0.0067)
		Central	0.0134(±0.0169)**	0.0063(±0.0089)
	C-S	Central	0.0444(±0.0407)***	0.0065(±0.0102)
		Southwest	0.0722(±0.0689)***	0.0172(±0.0216)
ln (Fixed +1) (%) (ancestral-state bases)	N-C	North	0.0564(±0.0379)▲▲▲	0.0530(±0.0246)▲▲▲
		Central	0.0883(±0.0410)** ▲▲▲	0.0662(±0.0288)▲▲▲
	C-S	Central	0.1004(±0.0575)*** ▲▲▲	0.0659(±0.0290)▲▲▲
		Southwest	0.1897(±0.0831)*** ▲▲▲	0.1146(±0.0465)▲▲▲
ln (Shared +1) (%)	N-C		0.2300(±0.0318)**	0.2462(±0.0184)
	C-S		0.1158(±0.0596)***	0.2138(±0.0342)
F_{ST}	N-C		0.4827(±0.0872)***	0.0769(±0.0558)
	C-S		0.7504(±0.0503)***	0.2038(±0.1318)
ln (d_{xy} +1)	N-C		0.1792(±0.1498)	0.1762(±0.1564)
	C-S		0.2888(±0.2276)	0.2637(±0.2090)

Notes: * indicate significant differences between outliers and background genomic regions, ▲ indicate significant differences between ancestral-state bases and derived bases, by Mann-Whitney U test (*/▲ *P*-value < 0.05; **/▲▲ *P*-value < 1e-4; ***/▲▲▲ *P*-value < 2.2e-16).

Table S5. List of genes located in a region of significantly high genetic differentiation between the north and central populations of *Populus davidiana*.

Poplar gene	Best Arabidopsis hit	Synonyms	Annotated description
Potri.001G015300	AT3G45140	LOX2	lipoxygenase 2
Potri.001G058100	AT1G27290		
Potri.001G170200	AT1G17460	TRFL3	TRF-like 3
Potri.001G284301	AT2G14210	ANR1	AGAMOUS-like 44
Potri.001G327100	AT3G27010	TCP20	TEOSINTE BRANCHED 1, cycloidea, PCF (TCP)-domain family protein 20
Potri.001G328300	AT3G01960	F1C9.26	
Potri.001G328600			
Potri.002G105500	AT2G01710		Chaperone DnaJ-domain superfamily protein
Potri.002G105600			
Potri.002G112300			
Potri.002G112400			
Potri.002G112500	AT5G17870	PSRP6	plastid-specific 50S ribosomal protein 6
Potri.002G112600	AT1G10020		Protein of unknown function (DUF1005)
Potri.002G128500	AT2G26530	AR781	Protein of unknown function (DUF1645)
Potri.002G128700	AT5G36210	MAB16.20	alpha/beta-Hydrolases superfamily protein
Potri.002G128900			
Potri.002G129000	AT4G09680	CTC1	conserved telomere maintenance component 1
Potri.002G129100			
Potri.002G129166	AT1G47720	OSB1	Primosome PriB/single-strand DNA-binding
Potri.002G180800	AT1G01060	LHY	Homeodomain-like superfamily protein
Potri.002G181000	AT3G61960	ATG1A	Protein kinase superfamily protein
Potri.002G260100			
Potri.004G123900	AT3G28720		(1 of 1) PTHR31515:SF3 - GENOMIC DNA, CHROMOSOME 3, BAC CLONE: T19N8
Potri.004G124000	AT3G28730	SSRP1	high mobility group
Potri.005G091700	AT5G23210	SCPL34	serine carboxypeptidase-like 34
Potri.005G091800			
Potri.005G131500	AT5G60150		
Potri.005G187300	AT1G21460	SWEET1	Nodulin MtN3 family protein
Potri.006G083900	AT5G02540		NAD(P)-binding Rossmann-fold superfamily protein
Potri.006G166300	AT5G57360	ADO1	Galactose oxidase/kelch repeat superfamily protein

Potri.006G166400	AT2G18910		hydroxyproline-rich glycoprotein family protein
Potri.006G166500	AT2G18900		Transducin/WD40 repeat-like superfamily protein
Potri.006G166800			
Potri.006G234500	AT5G11700		(1 of 2) PTHR31513:SF1 - GLYCINE-RICH PROTEIN
Potri.006G234600	AT3G05230		Signal peptidase subunit
Potri.006G238566			
Potri.007G031200	AT2G18630		Protein of unknown function (DUF677)
Potri.010G078500	AT5G43720	MQD19.5	Protein of unknown function (DUF2361)
Potri.010G078600	AT5G02940		Protein of unknown function (DUF1012)
Potri.010G079700			
Potri.010G144400	AT3G17030		Nucleic acid-binding proteins superfamily
Potri.010G144500	AT3G66654	CYP21-4	Cyclophilin-like peptidyl-prolyl cis-trans isomerase family protein
Potri.010G176300	AT3G12680	HUA1	floral homeotic protein (HUA1)
Potri.010G176400	AT1G54350	ABCC2	ABC transporter family protein
Potri.010G176600	AT1G79580	SMB	NAC (No Apical Meristem) domain transcriptional regulator superfamily protein
Potri.010G180300	AT3G48480		Cysteine proteinases superfamily protein
Potri.010G180400	AT5G36790	PGLP1B	Haloacid dehalogenase-like hydrolase (HAD) superfamily protein
Potri.010G180500			
Potri.011G150700	AT1G32990	RPL11	plastid ribosomal protein l11
Potri.011G150800			
Potri.013G138900	ATCG00900	rps7-A	Ribosomal protein S7p/S5e family protein
Potri.013G142232	ATCG00190	rpoB	RNA polymerase subunit beta
Potri.014G095500	AT2G46370	JAR1	Auxin-responsive GH3 family protein
Potri.014G180100	AT3G60160	ABCC9	multidrug resistance-associated protein 9
Potri.016G106400			
Potri.017G044600	AT5G64360	EIP9	Chaperone DnaJ-domain superfamily protein
Potri.018G090400	AT3G11710		lysyl-tRNA synthetase 1
Potri.018G090700	AT2G18950	HPT1	homogentisate phytyltransferase 1
Potri.018G090800	AT5G57360	ADO1	Galactose oxidase/kelch repeat superfamily protein

Table S6. List of genes located in a region of significantly high genetic differentiation between the central and southwest populations of *Populus davidiana*.

Poplar gene	Best Arabidopsis hit	Synonyms	Annotated description
Potri.001G065900	AT2G33530	SCPL46	serine carboxypeptidase-like 46
Potri.001G066000	AT1G28100	NDX1	
Potri.001G086700			
Potri.001G086800	AT1G64600		methyltransferases;copper ion binding
Potri.001G086900	AT4G24050	T19F6.14	NAD(P)-binding Rossmann-fold superfamily protein
Potri.001G087100	AT4G24040	TRE1	trehalase 1
Potri.001G132900	AT5G45560	EDR2L	Pleckstrin homology (PH) domain-containing protein / lipid-binding START domain-containing protein
Potri.001G148100	AT5G47010	UPF1	RNA helicase, putative
Potri.001G148200			
Potri.001G148300	AT5G47020	MQD22.16	
Potri.001G169700	AT3G13970	ATG12B	Ubiquitin-like superfamily protein
Potri.001G169900	AT1G17440	TAF12B	Transcription initiation factor TFIID subunit A
Potri.001G191400	AT3G16370	APG	GDSL-like Lipase/Acylhydrolase superfamily protein
Potri.001G191500	AT3G16380	PAB6	poly(A) binding protein 6
Potri.001G216400	AT5G22100		RNA cyclase family protein
Potri.001G216500	AT5G22120		(1 of 1) PTHR21678 - GROWTH INHIBITION AND DIFFERENTIATION RELATED PROTEIN 88
Potri.001G266000			
Potri.001G278800			
Potri.001G278900	AT2G30060	RANBP1B	Pleckstrin homology (PH) domain superfamily protein
Potri.001G285500			
Potri.001G285600	AT5G64420	MSJ1.26	DNA polymerase V family
Potri.001G326800	AT5G14080		Tetratricopeptide repeat (TPR)-like superfamily protein
Potri.001G327000	AT3G01990	ACR6	ACT domain repeat 6
Potri.001G328300	AT3G01960	F1C9.26	
Potri.001G328600			
Potri.001G331500	AT5G40680	MNF13.24	Galactose oxidase/kelch repeat superfamily protein
Potri.001G402500	AT1G52980	NUG2	GTP-binding family protein
Potri.001G402600			

Potri.001G402800	AT3G15430	MJK13.9	Regulator of chromosome condensation (RCC1) family protein
Potri.002G055000			
Potri.002G055100	AT3G59030	DTX41	MATE efflux family protein
Potri.002G110800			
Potri.002G110900	AT4G24470	GATA25	GATA-type zinc finger protein with TIFY domain
Potri.002G111000	AT1G58230	BCHB	binding
Potri.002G112100	AT5G49630	AAP6	amino acid permease 6
Potri.002G113800	AT1G58520	RXW8	lipases;hydrolases, acting on ester bonds
Potri.002G113900			
Potri.002G128700	AT5G36210	MAB16.20	alpha/beta-Hydrolases superfamily protein
Potri.002G143000	AT1G02080		transcription regulators
Potri.002G152100	AT2G42010	PLDBETA1	phospholipase D beta 1
Potri.002G157200	AT1G01790	KEA1	K+ efflux antiporter 1
Potri.002G201800	AT1G54970	PRP1	proline-rich protein 1
Potri.002G201900	AT2G47540		Pollen Ole e 1 allergen and extensin family protein
Potri.002G202000			
Potri.002G232600	AT2G31660	SAD2	ARM repeat superfamily protein
Potri.003G096100	AT5G46910	MQD22.4	Transcription factor jumonji (jmj) family protein / zinc finger (C5HC2 type) family protein
Potri.003G120000	AT1G63000	NRS/ER	nucleotide-rhamnose synthase/epimerase-reductase
Potri.003G120101			
Potri.003G131300	AT5G41410	BEL1	POX (plant homeobox) family protein
Potri.003G131400	AT4G11350		Protein of unknown function (DUF604)
Potri.004G134200	AT5G08480	VQ31	VQ motif-containing protein
Potri.004G134300	AT5G08490	PCMP-E32	Tetratricopeptide repeat (TPR)-like superfamily protein
Potri.004G134400			
Potri.004G134500	AT5G23575		Transmembrane CLPTM1 family protein
Potri.004G201800	AT2G27460		sec23/sec24 transport family protein
Potri.004G235700			
Potri.004G235800	AT2G35610	XEG113	xyloglucanase 113
Potri.004G235900	AT4G19210	ABCE2	RNAse l inhibitor protein 2
Potri.004G236000	AT3G07530	F21O3.24	(1 of 1) PTHR11203:SF2 - INTEGRATOR COMPLEX SUBUNIT 9
Potri.004G236300	AT4G19190	PCMP-E1	zinc knuckle (CCHC-type) family protein
Potri.004G236500	AT1G31770	ABCG14	ATP-binding cassette 14
Potri.004G236600	AT1G31780	COG6	(1 of 1) PTHR21506 - COMPONENT OF OLIGOMERIC GOLGI COMPLEX 6

Potri.004G236900	AT4G19180	APY7	GDA1/CD39 nucleoside phosphatase family protein
Potri.004G237000			
Potri.005G078600			
Potri.005G091600	AT5G08280	HEMC	hydroxymethylbilane synthase
Potri.005G093000	AT5G23110	MYJ24.15	Zinc finger, C3HC4 type (RING finger) family protein
Potri.005G093100	AT3G18610	NUCL2	nucleolin like 2
Potri.005G126600			
Potri.005G126700	AT5G09380		RNA polymerase III RPC4
Potri.005G151200			
Potri.005G190200			
Potri.005G196400	AT4G09830		Uncharacterised conserved protein UCP009193
Potri.005G196500	AT1G34020	URGT6	Nucleotide-sugar transporter family protein
Potri.005G197000	AT1G71696	SOL1	carboxypeptidase D, putative
Potri.005G197200			
Potri.005G210300	AT4G02780	GA1	Terpenoid cyclases/Protein prenyltransferases superfamily protein
Potri.005G225900	AT2G20495		(1 of 1) PTHR15243 - SERINE/THREONINE-PROTEIN KINASE 19
Potri.005G226000	AT3G23300		S-adenosyl-L-methionine-dependent methyltransferases superfamily protein
Potri.005G256700	AT1G20880		RNA-binding (RRM/RBD/RNP motifs) family protein
Potri.005G257000	AT1G76490	HMG1	hydroxy methylglutaryl CoA reductase 1
Potri.005G257100	AT2G33585		
Potri.006G028500			
Potri.006G132100	AT2G37050	LRR-RLK	Leucine-rich repeat protein kinase family protein
Potri.006G143300	AT2G06210	VIP6	binding
Potri.006G145100	AT1G17590	NFYA8	nuclear factor Y, subunit A8
Potri.006G162900	AT4G28910	AFPH2	novel interactor of JAZ
Potri.006G163900	AT5G19680		Leucine-rich repeat (LRR) family protein
Potri.006G164000	AT2G20000	CDC27B	CDC27 family protein
Potri.006G166300	AT5G57360	ADO1	Galactose oxidase/kelch repeat superfamily protein
Potri.006G166400	AT2G18910		hydroxyproline-rich glycoprotein family protein
Potri.006G166500	AT2G18900		Transducin/WD40 repeat-like superfamily protein
Potri.006G208800	AT3G11550	CASP2	Uncharacterised protein family (UPF0497)

Potri.006G218100	AT5G21970		Ubiquitin carboxyl-terminal hydrolase family protein
Potri.006G238300			
Potri.006G238400	AT2G25830		YebC-related
Potri.006G238500	AT4G32810	CCD8	carotenoid cleavage dioxygenase 8
Potri.007G029300	AT4G36720	HVA22K	HVA22-like protein K
Potri.007G029400	AT4G36730	GBF1	G-box binding factor 1
Potri.007G055900	AT3G49910	RPL26A	Translation protein SH3-like family protein
Potri.007G057000	AT4G35987	CaMKMT	S-adenosyl-L-methionine-dependent methyltransferases superfamily protein
Potri.007G145100	AT2G31830	IP5P14	endonuclease/exonuclease/phosphatase family protein
Potri.007G145200	AT1G05640		Ankyrin repeat family protein
Potri.007G147100	AT3G59950	ATG4B	Peptidase family C54 protein
Potri.007G147200	AT2G44150	ASHH3	histone-lysine N-methyltransferase ASHH3
Potri.008G036300	AT2G39130	AVT1C	Transmembrane amino acid transporter family protein
Potri.008G036400	AT5G04260	WCRKC2	WCRKC thioredoxin 2
Potri.008G036500	AT3G10360	APUM4	pumilio 4
Potri.008G077700	AT4G20370	TSF	PEBP (phosphatidylethanolamine-binding protein) family protein
Potri.008G077800	AT1G65470	FAS1	chromatin assembly factor-1 (FASCIATA1) (FAS1)
Potri.008G116800	AT1G68830	STN7	STT7 homolog STN7
Potri.008G116900	AT1G13250	GATL3	galacturonosyltransferase-like 3
Potri.008G134600	AT2G01690	VAC14	ARM repeat superfamily protein
Potri.008G136300	AT1G68040		S-adenosyl-L-methionine-dependent methyltransferases superfamily protein
Potri.008G143700	AT2G02148		(1 of 1) PTHR13555 - C2H2 ZINC FINGER CGI-62-RELATED
Potri.008G144000			
Potri.008G159400	AT3G63410	VTE3	S-adenosyl-L-methionine-dependent methyltransferases superfamily protein
Potri.008G159500	AT3G55760	F1I16_170	
Potri.009G016900	AT1G08420	BSL2	BRI1 suppressor 1 (BSU1)-like 2
Potri.009G033100			
Potri.009G043800	AT2G29650	ANTR1	phosphate transporter 4;1
Potri.010G081300	AT5G43810	AGO10	Stabilizer of iron transporter SufD / Polynucleotidyl transferase
Potri.010G091200	AT5G19310	CHR23	Homeotic gene regulator
Potri.010G091400	AT5G19300		(1 of 1) PTHR12150:SF13 - DUF171 FAMILY PROTEIN
Potri.010G092200	AT5G19210	RH58	P-loop containing nucleoside triphosphate

			hydrolases superfamily protein
Potri.010G092300	AT4G14220	RHF1A	RING-H2 group F1A
Potri.010G092800			
Potri.010G092900	AT1G69830	AMY3	alpha-amylase-like 3
Potri.010G115200	AT1G71090	PILS2	Auxin efflux carrier family protein
Potri.010G115500	AT1G71110		
Potri.010G116300			
Potri.010G116400	AT1G15130	BRO1	Endosomal targeting BRO1-like domain-containing protein
Potri.010G138900	AT1G73430	COG3	sec34-like family protein
Potri.010G143600			
Potri.010G143700			
Potri.010G144400	AT3G17030		Nucleic acid-binding proteins superfamily
Potri.010G144500	AT3G66654	CYP21-4	Cyclophilin-like peptidyl-prolyl cis-trans isomerase family protein
Potri.010G154100	AT1G26310	CAL	K-box region and MADS-box transcription factor family protein
Potri.010G195000	AT3G55730	F1I16_140	myb domain protein 109
Potri.010G209300	AT5G13660		(1 of 18) PF16135 - TPL-binding domain in jasmonate signalling (Jas)
Potri.011G057000	AT1G28370	ERF11	ERF domain protein 11
Potri.011G082800	AT5G46180	DELTA-OAT	ornithine-delta-aminotransferase
Potri.011G169200	AT4G26450	WIP1	
Potri.012G062300			
Potri.012G063400	AT5G08100	At5g08100	N-terminal nucleophile aminohydrolases (Ntn hydrolases) superfamily protein
Potri.012G063800	AT5G60850	DOF5.4	OBF binding protein 4
Potri.012G064100	AT4G37120	At4g37120	Pre-mRNA splicing Prp18-interacting factor
Potri.012G064200			
Potri.012G066900			
Potri.012G067000	AT5G07810	MXM12.5	SNF2 domain-containing protein / helicase domain-containing protein / HNH endonuclease domain-containing protein
Potri.012G067800	AT1G74330		Protein kinase superfamily protein
Potri.013G004801	AT5G26749		C2H2 and C2HC zinc fingers superfamily protein
Potri.013G004901			
Potri.013G005000			
Potri.013G047500	AT3G04470		Ankyrin repeat family protein
Potri.013G047600	AT5G28960		(1 of 2) PTHR13132 - ALPHA- 1,6 -FUCOSYLTRANSFERASE
Potri.013G056500	AT3G13540	MYB5	myb domain protein 5

Potri.013G065700	AT3G03710	PNP1	polyribonucleotide nucleotidyltransferase, putative
Potri.013G081300			
Potri.014G016900	AT1G27752		Ubiquitin system component Cue protein
Potri.014G017000	AT1G27750		nucleic acid binding
Potri.014G028900	AT5G09890	MYH9.10	Protein kinase family protein
Potri.014G029000	AT5G42900	COR27	cold regulated gene 27
Potri.014G140900	AT5G36880	ACS	acetyl-CoA synthetase
Potri.014G141000	AT1G12920	ERF1-2	eukaryotic release factor 1-2
Potri.014G148800	AT2G32000		DNA topoisomerase, type IA, core
Potri.015G088900	AT3G48425	APE1L	DNAse I-like superfamily protein
Potri.015G089000	AT3G48430	REF6	relative of early flowering 6
Potri.015G089300	AT2G36850	CALS10	glucan synthase-like 8
Potri.017G129000	AT5G37850	PK	pfkB-like carbohydrate kinase family protein
Potri.018G046700	AT2G25740		ATP-dependent protease La (LON) domain protein
Potri.018G064700	AT1G17590	NFYA8	nuclear factor Y, subunit A8
Potri.018G064800	AT5G20520	WAV2	alpha/beta-Hydrolases superfamily protein
Potri.019G068200			
Potri.019G068300			
